# Comparison of the Mediterranean Diet and Other Therapeutic Strategies in Metabolic Syndrome: A Systematic Review and Meta-Analysis

**DOI:** 10.3390/ijms26125887

**Published:** 2025-06-19

**Authors:** Alejandro Bruna-Mejias, Jessica San Martin, Danna Arciniegas-Diaz, Trinidad Meneses-Caroca, Amelia Salamanca-Cerda, Antonia Beas-Gambi, Jessica Paola-Loaiza-Giraldo, Cynthia Ortiz-Ahumada, Pablo Nova-Baeza, Gustavo Oyanedel-Amaro, Mathias Orellana-Donoso, Alejandra Suazo-Santibáñez, Juan Sanchis-Gimeno, Juan José Valenzuela-Fuenzalida

**Affiliations:** 1 Departamento de Ciencias y Geografía, Facultad de Ciencias Naturales y Exactas, Universidad de Playa Ancha, Valparaíso 2360072, Chile; alejandro.bruna@upla.cl; 2 Departamento de Morfología, Facultad de Medicina, Universidad Andrés Bello, Santiago 8370146, Chile; jessicasanmartinp@gmail.com (J.S.M.); arcidanna2005@gmail.com (D.A.-D.); trini.meneses46@gmail.com (T.M.-C.); ameliasalamancacerda@gmail.com (A.S.-C.); aantoobeas@gmail.com (A.B.-G.); cybepaorah@gmail.com (C.O.-A.); pablo.nova@usach.cl (P.N.-B.); 3 Facultad de Ciencias de la Salud, Unidad Central del Valle del Cauca (UCEVA), Tuluá 763022, Valle del Cauca, Colombia; yessica.loaiza01@uceva.edu.co; 4 Facultad de Ciencias de la Salud, Universidad Autónoma de Chile, Santiago 7500912, Chile; g.oyanedelamaro@gmail.com; 5 Escuela de Medicina, Universidad Finis Terrae, Santiago 7501015, Chile; mathor94@gmail.com; 6 Faculty of Medicine and Science, Universidad San Sebastián, Santiago 8420524, Chile; 7 Faculty of Health and Social Sciences, Universidad de Las Américas, Santiago 8370040, Chile; alej.suazo@gmail.com; 8 GIAVAL Research Group, Department of Anatomy and Human Embryology, Faculty of Medicine, University of Valencia, 46001 Valencia, Spain; juan.sanchis@uv.es; 9 Departamento de Ciencias Química y Biológicas, Facultad de Ciencias de la Salud, Universidad Bernardo O’Higgins, Santiago 8370993, Chile

**Keywords:** mediterranean diet, mediterranean food, eat mediterranean, metabolic syndrome, x syndrome

## Abstract

The Mediterranean diet (MD) is one of the healthiest diets, high in fiber, antioxidants, and unsaturated fats. MD improves lipid profiles, reduces inflammation, controls blood pressure, decreases insulin resistance, and enhances the sensitivity to this hormone, lowering the risks of Metabolic syndrome (MS). MS is characterized by central obesity, hypertension, insulin resistance, and dyslipidemia, increasing the risk of cardiovascular disease and type II diabetes. The objective of this study was to know the effectiveness of the MD versus other treatments in patients with MS. A systematic search across multiple databases, Medline, Embase, Web of Science, Scopus, Google Scholar, and Cinahl, was conducted using keywords such as “Mediterranean diet”, “Mediterranean food”, “eat mediterranean”, “Metabolic syndrome”, and “x syndrome”. A total of 12 studies met the inclusion criteria. Mediterranean diet at different doses versus other diets or other treatments showed significant improvements in clinical parameters, including BMI (mean difference of −0.83 95% CI: −0.93 to −0.74; *p* < 0.00001),waist circumference (mean difference = −1.81, CI = −2.63 to −0.99, *p* < 0.00001) triglycerides (mean difference = −22.38, CI = −32.86 to −11.90, *p* < 0.00001), Glucose (mean difference = −4.28, CI = −7.64 to −0.93, *p* = 0.005) and, HOMA IR (mean difference = −0.72, CI = −0.78 to −0.65, *p* < 0.00001), and Insulin resistance (mean difference = −2.98, CI = −3.27 to −2.69, *p* < 0.00001), all of which improved, Although there were more outcomes, these are the most important changes for patients with metabolic syndrome. MD improves metabolic and cardiovascular health, but study heterogeneity limits the results’ generalizability. Because of that, further research is needed to standardize approaches and explore their mechanisms. MD should be part of an optimized strategy that includes education and physical activity. The strength of the evidence was very low according to the GRADE approach. Further research is needed to support the efficacy of the Mediterranean diet in patients with MS.

## 1. Introduction

The Mediterranean diet (MD) has become one of the healthiest currently available due to its numerous health benefits [1,2]. It is characterized by high consumption of fruits and vegetables, which provide vitamins, fiber, and minerals. Legumes are essential sources of protein and fiber, while nuts and seeds are rich in unsaturated fats and antioxidants. Cereals, primarily whole grains, provide vital nutrients and energy [3]. In this diet, the majority of fat—around 70–80%—comes from extra virgin olive oil, which is the main source and provides a high content of unsaturated fats [4]. Consumption of red and white meat is moderate to low and is limited to lunch. Dairy products such as cheese or yogurt and products with a high sugar content are also consumed in smaller quantities [5]. Moderate alcohol consumption is recommended, preferably wine to accompany meals [6]. Thanks to its high content of unsaturated fats and other healthy components, this diet has been shown to have significant benefits. This dietary pattern improves lipid profile, decreases LDL cholesterol levels, and helps control high blood pressure [3]. It also reduces inflammation and oxidative stress levels due to the high consumption of antioxidants in vegetables and nuts [4]. It also improves body composition by lowering adiposity and increasing muscle mass [7,8]. In addition, it allows a decrease in insulin resistance and an increase in sensitivity to this hormone [9]. It has even been associated with a lower incidence of neurodegenerative diseases and certain types of cancer since its components favor more anti-inflammatory profiles [4]. Adherence to this type of diet has been associated with a reduction in the prevalence and incidence of metabolic syndrome (MS) since it positively impacts the reduction of waist circumference, the decrease in triglyceride levels, the increase in HDL cholesterol, and the reduction of blood pressure, all of these represent cardiometabolic risk factors.

The presence of various metabolic abnormalities, such as central obesity, insulin resistance, arterial hypertension, and dyslipidemia, defines MS. This condition significantly increases the risk of developing atherosclerotic cardiovascular diseases and type II diabetes mellitus [10]. The diagnosis of MS is confirmed with the presence of three or more of these metabolic abnormalities, characterized by the following values: waist circumference > 101.6 cm (men) and >88.9 cm (women); serum triglycerides ≥ 150 mg/dL; HDL < 40 mg/dL (men) and <50 mg/dL (women); fasting glucose > 100 mg/dL; systolic pressure > 130 mmHg and diastolic pressure > 85 mmHg. This highlights the need to adopt proactive strategies for early identification and appropriate intervention. The etiology of MS is complex and multifactorial [11]. The factors involved include genetic predisposition and environmental or lifestyle-related elements, such as obesity, lack of physical activity, and an unhealthy diet. The main feature of the syndrome is the accumulation of fat, especially in the abdominal region, leading to insulin resistance. Excessive adipose tissue releases proinflammatory cytokines, such as tumor necrosis factor, leptin, adiponectin, plasminogen activator inhibitor, and resistine, which impair and aggravate insulin resistance. The prevalence of this syndrome in the Western population ranges from 20% to 40%, being more common in women. Various studies indicate that adequate management of MS requires lifestyle changes, primarily weight loss through diet and exercise. Pharmacological treatment should be considered for those people whose risk factors are not adequately reduced with the indicated measures [12,13,14]. It is important to highlight that, although the included studies refer to the “Mediterranean Diet”, relevant variations in nutritional composition were observed across different demographic groups. These differences may be attributed to factors such as geographical location, regional dietary habits, age, sex, body weight, or differing methodological approaches. These variations should be taken into account when interpreting the evidence and comparing the effects attributed to the Mediterranean diet.

The aim of this study was to assess the effectiveness of the MD compared to other treatments in patients with MS.

## 2. Methods

### 2.1. Prisma Guidelines and PROSPERO Registration

This systematic review and meta-analysis were performed and documented following the guidelines outlined in the Preferred Reporting Items for Systematic Reviews and Meta-Analyses (PRISMA) statement [10]. The study is registered under number CRD42025638166 in the International Prospective Register of Systematic Reviews (PROSPERO).

### 2.2. Literature Search

To identify relevant literature, we conducted a systematic search across electronic databases, including MEDLINE (via PubMed), EMBASE, SCOPUS, the Cochrane Central Register of Controlled Trials, the Cumulative Index to Nursing and Allied Health Literature, and Web of Science AND google scholar, encompassing records from their inception until February 2025. Our criteria for inclusion emphasized controlled or randomized clinical trials published in either English or Spanish. The search strategy employed various combinations of keywords such as “Mediterranean diet”, “Mediterranean food”, “eat Mediterranean”, “metabolic syndrome”, and “X syndrome.” The titles and abstracts of the retrieved references were independently evaluated by two authors (JSM and JJV-F). Full texts were acquired for any references considered potentially relevant by either author. In instances of disagreement, a third reviewer (TM) was consulted to achieve a consensus.

### 2.3. Data Extraction

Two researchers from the team (MB-V and ML-C) independently analyzed pertinent data for each trial. They examined various aspects of the studies, including the authors and publication year, study type, total participant count, outcomes, statistical values, key findings, geographical location, gender distribution, and the type and dosage of interventions. The methodological quality of the studies included was evaluated using the Cochrane Risk of Bias (RoB) tool [15], which assesses seven areas: random sequence generation, concealment of the randomization sequence, blinding of participants and treatments, blinding of outcome assessment, incomplete outcome data, selective reporting, and other potential biases. Each area was rated as having a “low”, “unclear”, or “high” risk of bias. Any disagreements were resolved through discussion, and if a consensus could not be achieved, a third reviewer (JJV-F) made the final determination. The level of agreement between the reviewers was measured using kappa statistics, which indicated a substantial agreement with a kappa value of 0.88.

### 2.4. Types Studies

This meta-analysis involved selecting studies based on specific criteria: participants diagnosed with MS or syndrome X, individuals who adhered to an MD through various methods, and evaluating outcomes such as metabolic markers, clinical indicators, and quality of life assessments. The analysis focused on randomized clinical trials and experimental research. Studies were excluded if they were editorials, case reports or series, literature reviews, or involved non-human subjects; if they included patients with other medical conditions; if they utilized treatments with other dier; or if they lacked a control group for comparison.

### 2.5. Data Analysis and Rating Quality of Evidence

Various scales were used for MS parameters and were analyzed as continuous variables. These included Body weight, body mass index (BMI), Waist Circumference, systolic blood pressure (SBP), diastolic blood pressure (DBP), Triglycerides, Total Cholesterol, LDL (Low-density lipoproteins), HDL (High-density lipoproteins), Insulin, and homeostatic model assessment for insulin resistance (HOMA-IR). The effect size was calculated using the mean difference, we have decided not to abbreviate average differences, since the Mediterranean diet abbreviates in the same way. Effect sizes were classified as trivial (<0.2), small (0.2–0.5), medium (0.6–0.8), and large (>0.8). The Hartung–Knapp–Sidik–Jonkman random effects model or the Mantel–Haenszel fixed effects method was applied to estimate the pooled effect size, depending on data heterogeneity. The MD values, with their respective 95% confidence intervals (CIs), ranged from 2 to −2. We assessed the heterogeneity of results across studies using the I^2^ statistic, interpreting values of 0 to 40% as “may not be important”, 30 to 60% as “moderate”, 50 to 90% as “substantial”, and 75 to 100% as “considerable” heterogeneity. Additionally, we visually examined the forest plots for overlapping CIs and the associated *p* values. The meta-analysis was performed using RevMan 5.4. The synthesis and quality of evidence for each outcome were assessed using the Grading of Recommendation, Assessment, Development, and Evaluation (GRADE) framework [16]. The quality of evidence was classified into four levels: high, moderate, low, and very low. We utilized the GRADE profiler to import data from RevMan 5.4 and create a “summary of findings” table, which can be found in Supplementary Table S3.

## 3. Results

### 3.1. Selection of Studies

**Figure 1:**
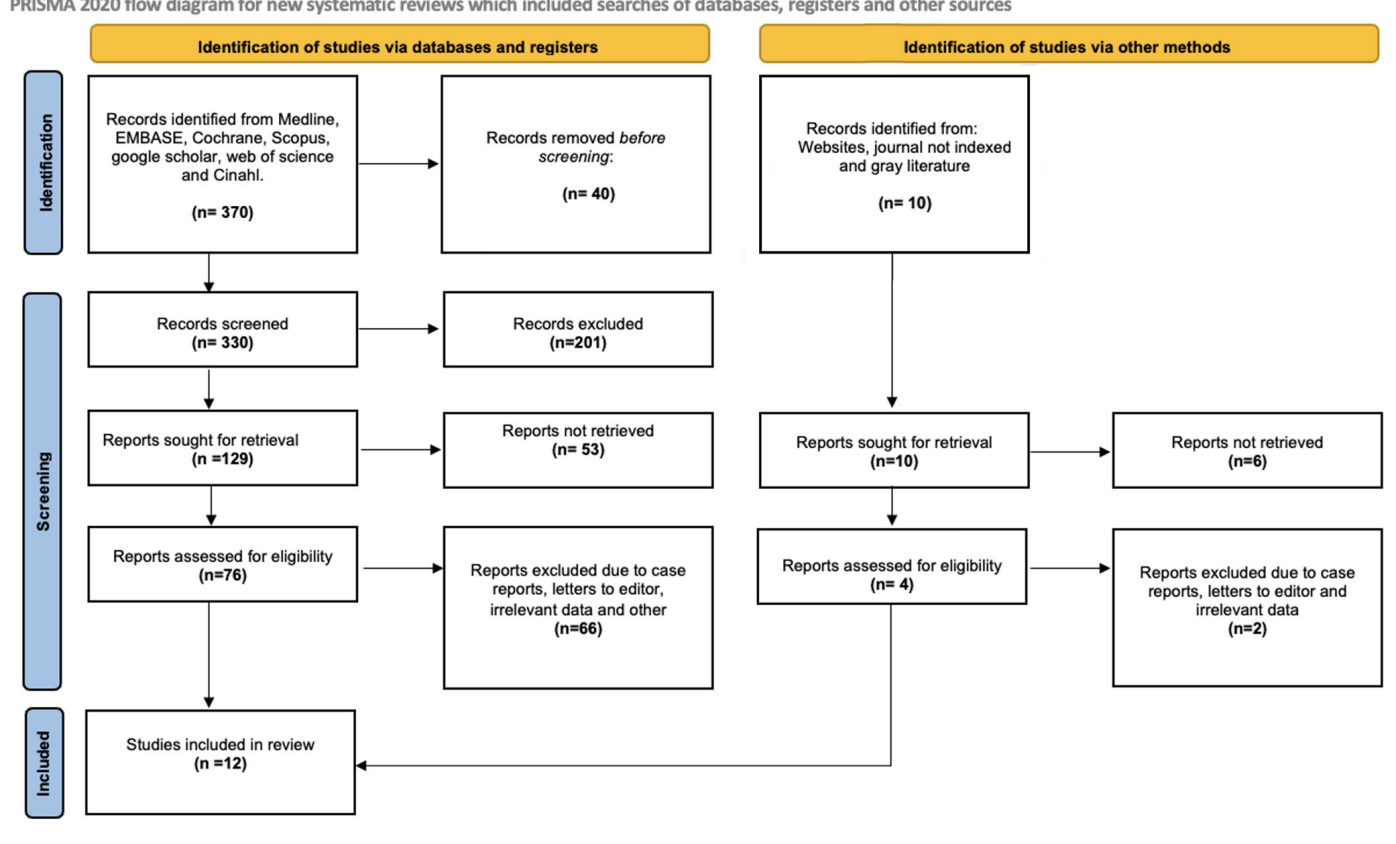
Flow diagram included studies.

A total of 370 articles were identified through the electronic search, with 86 deemed potentially eligible for full-text review. A flow diagram (Figure 1) illustrates the process of selecting articles for the systematic review. No additional studies were found through clinical trial registries. Ultimately, this review incorporated 12 randomized controlled trials that met the eligibility criteria.

### 3.2. Study Characteristics

The included studies are summarized in Table 1 and Table 2. For this study, twelve studies met the investigator’s inclusion criteria. The studies were published between 2005 and 2020. They were conducted in the USA, China, India, Puerto Rico, Iran, Japan, Argentina, Australia, Brazil, Canada, Chile, Taiwan, Hungary, Croatia, Poland, Germany, and the Russian Federation. The population of the included studies included 2013 patients (1272 in the MD group and 741 in the other therapeutic modalities group). The mean ages were 59 (±2.7) and 59.7 (±1.8) years for the MD and other therapeutic modalities groups, respectively. The mean duration of follow-up was 24.8 months (range 8 to 52). For the meta-analysis, only five studies could be grouped by outcomes, results, and treatment dose with MD [2,3,17,18,19]. The remaining seven studies [1,4,8,20,21,22,23], which could not be categorized by their results, indicated that MD enhanced various clinical parameters linked to MS.

### 3.3. Assessment of Risk of Bias in Individual Studies

**Figure 2:**
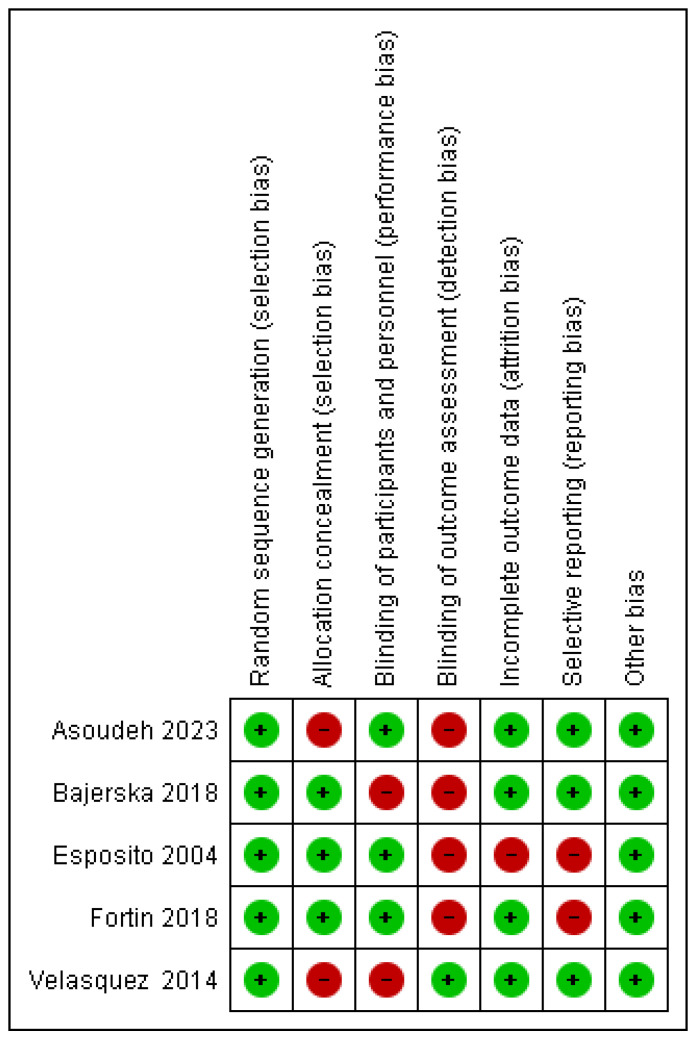
Risk of bias summary included studies [2,3,17,18,19].

**Figure 3:**
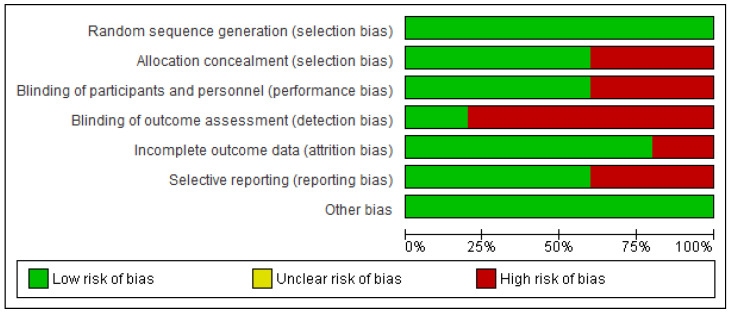
Diagram risk of bias of studies included [2,3,17,18,19].

Figure 2 and Figure 3 show the bias analysis using the Rob tool, which assessed seven domains that may represent construction bias in primary studies. In terms of random sequence generation, all studies (100%) were rated as “low risk” [2,3,17,18,19] of bias. All trials were considered to have a “low risk” of bias regarding other biases [2,3,17,18,19]. For incomplete outcome data, 80% of studies were rated as “low risk” [3,17,18,19], while the remaining 20% were identified as “high risk” [2]. Concerning the blinding of outcome assessment, 20% of trials were classified as “low risk” [19], whereas 80% exhibited a “high risk” of bias [2,3,17,18]. Similarly, for the blinding of participants and personnel, 60% of studies were assessed as having a “low risk” [2,3,18], while 40% were considered “high risk” [17,19]. Finally, regarding allocation concealment, 60% of trials were categorized as having a “low risk” of bias [2,17,18], whereas 40% were rated as “high risk” [3,19].

### 3.4. Synthesis of Results

The results of this meta-analysis were calculated using continuous variables in RevMan 5 software (Cochrane Collaboration). The total number of participants per group (*n*), the mean per group for each outcome, and the standard deviation (SD) were entered for each study. When the original study did not report the SD, it was estimated from the confidence interval using the following formula:

SD = √*n* × (Upper limit − Lower limit)/3.92

This formula is based on a statistical rule that relates the 95% confidence interval range to the standard deviation in normal distributions.

The inverse variance method was applied, in which a greater decrease in a variable (e.g., blood pressure or glucose) represents a positive clinical effect and shifts the diamond in the forest plot toward the experimental group.

All continuous outcome variables and their respective results are summarized in Table 3 and described in detail in the results sections below. Only these outcomes were included because they were the only ones that could be grouped across the studies analyzed.

#### 3.4.1. Body Weight

**Figure 4:**
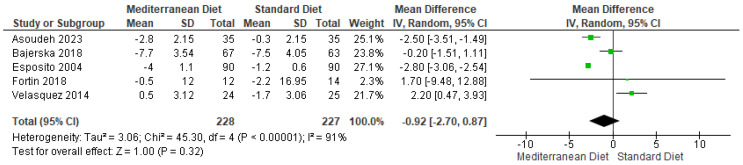
Forest plot of body weight in k/g [2,3,17,18,19].

Body weight is a parameter that should be analyzed in patients with MS. The pooled analysis indicated not statistically significant differences in the five studies evaluated (mean difference = −0.92; CI = −2.70 to 0.87; *p* = 0.32 (Figure 4) [2,3,17,18,19]. The direction of the effect was consistent; for this analysis, substantial heterogeneity was observed; in addition, it was presented in a statistically significant way between the studies (I^2^ = 91% and *p* < 0.00001. The overall certainty of this evidence, according to the GRADE rating, was classified as very low quality, so the results should be taken cautiously (Supplementary Table S3).

#### 3.4.2. Outcome BMI

**Figure 5:**
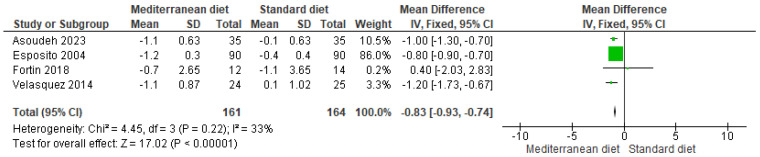
Forest plot of BMI in kg/m^2^ [2,3,18,19].

The comparison between the standard and Mediterranean diets showed a statistically significant reduction in body mass index (BMI) in favor of the Mediterranean diet. The meta-analysis, which included four studies, yielded a mean difference of −0.83 (95% CI: −0.93 to −0.74; *p* < 0.00001), indicating strong statistical significance (Figure 5) [2,3,18,19]. The direction of effect was consistent across the included studies. Although there was a slight overlap in the two confidence intervals, heterogeneity was low (I^2^ = 33%; *p* = 0.22), suggesting good consistency between the results. Nevertheless, according to the GRADE system, the overall quality of the evidence was classified as very low.

#### 3.4.3. Outcome Waist Circumference

**Figure 6:**
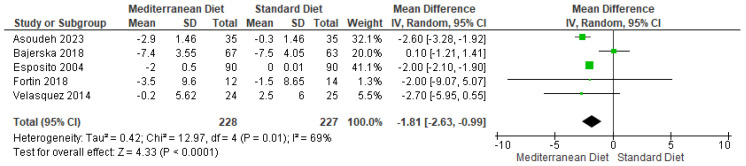
Forest plot of waist circumference in cm [2,3,17,18,19].

For the outcome of waist circumference, the standard diet was compared with the MD, and the analysis showed a statistically significant difference (mean difference = −1.81, CI = −2.63 to −0.99, *p* < 0.00001) (Figure 6) [2,3,17,18,19]. Regarding I^2^, substantial statistical heterogeneity was observed between the included studies for this outcome (I^2^ = 69% and *p* = 0.01). The certainty of this evidence was assessed using the GRADE rating and was determined to be of very low quality (Table S3).

#### 3.4.4. Outcome SBP

**Figure 7:**
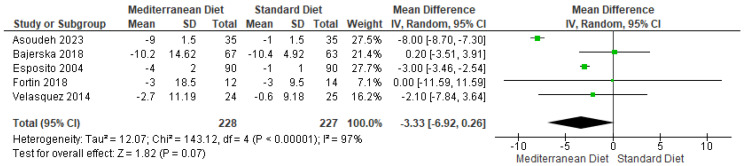
Forest plot of systolic blood pressure (SBP) in mmHg [2,3,17,18,19].

For the SBP outcome, when the standard diet was compared with the MD, it showed no statistically significant differences in all five trials assessed (Mean difference = −.3.33, CI = −6.92 to −0.26, *p* = 0.07 (Figure 7) [2,3,17,18,19]. Substantial statistical heterogeneity was observed between the included studies to analyze this outcome (I^2^ = 97% and *p* < 0.00001). The GRADE rating assessed the overall certainty of this evidence as very low quality (Table S3).

#### 3.4.5. Outcome DBP

**Figure 8:**
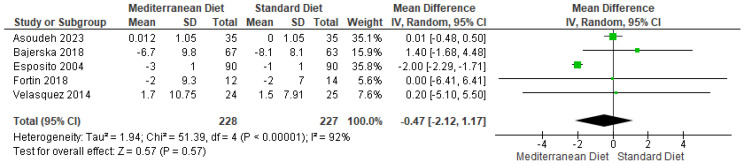
Forest plot of diastolic blood pressure (DBP) in mmHg [2,3,17,18,19].

For the DBP outcome, when comparing the standard diet with the MD, it showed a not statistically significant difference in the two evaluated trials (mean difference = −0.47; CI = −2.12 to 1.17; *p* = 0.57 (Figure 8) [2,3,17,18,19]). This analysis observed substantial heterogeneity between the studies (I^2^ = 96% and *p* < 0.00001). According to GRADE, the overall certainty of this evidence was rated as very low quality (Table S3).

#### 3.4.6. Outcome Triglycerides

**Figure 9:**
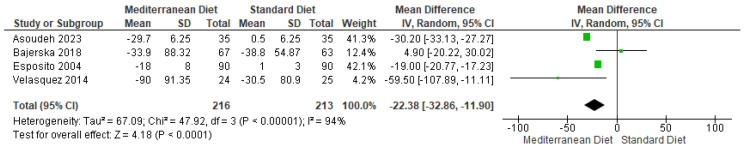
Forest plot of triglycerides in mg/dL [2,3,17,19].

For the triglyceride outcome, pooled analysis showed that participants displayed a statistically significant difference for this outcome (mean difference = −22.38, CI = −32.86 to −11.90, *p* < 0.00001) (Figure 9) [2,3,17,19]. Substantial heterogeneity was observed between the included studies assessing the triglyceride outcome (I^2^ = 94% and *p* < 0.00001). According to the GRADE rating, the overall certainty of the evidence was classified as very low quality (Supplementary Table S3).

#### 3.4.7. Outcome Glucose

**Figure 10:**
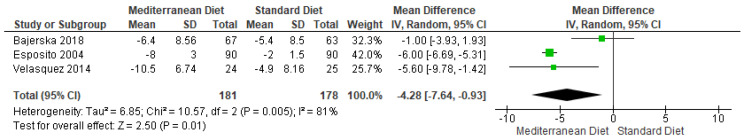
Forest plot of glucose in mg/dL [2,17,19].

The comparison between the standard treatment diet and the MD for blood glucose outcomes revealed a statistically significant difference favoring the MD group (mean difference = −4.28, CI = −7.64 to −0.93, *p* = 0.005) (Figure 10) [2,17,19]. There was considerable heterogeneity between the included studies for this outcome (I^2^ = 81% and *p* = 0.005). Based on the GRADE assessment, the overall quality of this evidence was graded as very low quality (Table S3).

#### 3.4.8. Outcome Total Cholesterol

**Figure 11:**
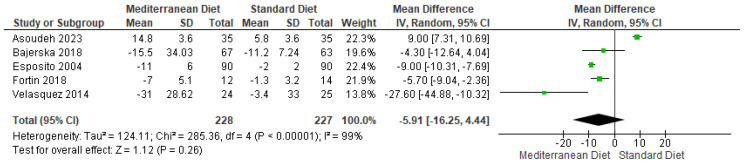
Forest plot of total cholesterol in mg/dL [2,3,17,18,19].

A comparison between the standard diet and the MD for total cholesterol outcomes revealed a statistically significant difference favoring the MD (mean difference = −5.91, CI = −16.25 to −4.44, *p* = 0.26) (Figure 11) [2,3,17,18,19]. Heterogeneity was statistically significant in the included studies for this outcome (I^2^ = 99% and *p* < 0.00001). The evidence’s overall quality was very low according to the GRADE rating (Table S3).

#### 3.4.9. Outcome HDL

**Figure 12:**
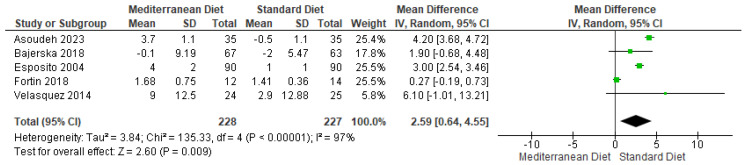
Forest plot of HDL in mg/dL [2,3,17,18,19].

For the HDL outcome, evidence from the pooled analysis showed that there was a statistically significant difference in favor of MD for this outcome (mean difference = 2.59, CI = 0.64 to 4.55, *p* = 0.009 (Figure 12) [2,3,17,18,19]. There was a substantial statistical heterogeneity between the included studies for this outcome (I^2^ = 97% and *p* < 0.00001). The GRADE assessment rated the overall quality of the evidence as very low (Table S3).

#### 3.4.10. Outcome LDL

**Figure 13:**
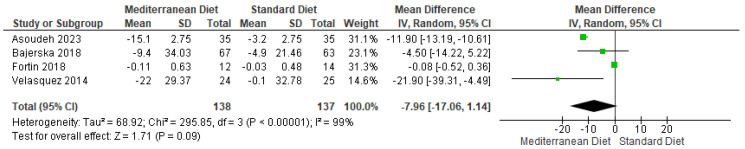
Forest plot of LDL in mg/dL [3,17,18,19].

Not Statistically significant differences in the LDL outcome were observed in the pooled analysis, with results favoring the MD (mean difference = −7.69, CI = −17.06 to −1.14, *p* < 0.00001) (Figure 13) [3,17,18,19]. There was substantial heterogeneity between studies (I^2^ = 99% and *p* < 0.00001). Based on the GRADE assessment, the overall certainty of this evidence was assessed as very low (Table S3).

#### 3.4.11. Body Fat

**Figure 14:**
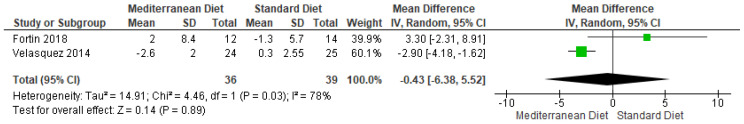
Forest plot of body fat in kg [18,19].

The analysis of body fat outcomes in the two assessed studies revealed not statistically significant differences between participants (mean difference = −0.43, CI = −6.38 to 5.52, *p* < 0.0001) (Figure 14) [18,19]. There was substantial statistical heterogeneity between studies (I^2^ = 78% and *p* = 0.03). Based on the GRADE rating, the overall certainty of the evidence was determined to be of very low quality (Table S3).

#### 3.4.12. HOMA-IR

**Figure 15:**
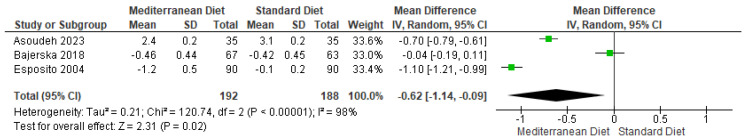
Forest plot of HOMA-IR [2,3,17].

When comparing the standard diet with the MD, pooled analysis showed no significant differences between participants in the three assessed studies (mean difference = −0.62; CI = −1.14 to −0.09; *p* = 0.02 (Figure 15) [2,3,17]. Substantial heterogeneity was observed between studies (I^2^ = 98% and *p* < 0.00001. Based on the GRADE assessment, the overall quality of the evidence was considered very low (Table S3).

#### 3.4.13. Insulin

**Figure 16:**
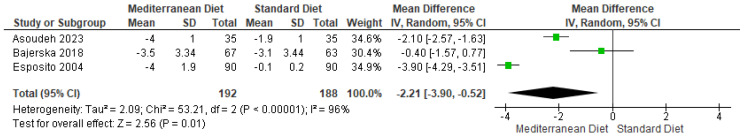
Forest plot of insulin in μU/mL [2,3,17].

For the insulin outcome, it showed statistically significant differences between the three studies analyzed (mean difference = −2.21, CI = −3.90 to −3.51, *p* = 0.01) (Figure 16) [2,3,17]. Substantial statistical heterogeneity was observed between the studies (I^2^ = 96% and *p* < 0.00001). The GRADE rating determined that the overall certainty of this evidence was of very low quality (Table S3).

## 4. Adverse Effects

Not all included studies reported on the type of adverse effect or safety risk in patients with MS. For this purpose, five studies [2,3,17,18,19] analyzed whether the dietary diet presented any type of adverse effect in patients with MS, showing as a main result that no attributable adverse effects were reported, which shows its safety profile as a sustainable and healthy dietary intervention, but since this is a limited number of studies, we could not ensure that the MD will always be safe and for all types of patients.

## 5. Meta-Regression

The main results of the meta-regression analyses with moderators of age, calories consumed, how many times a week the diet was used, time of the diet in weeks, the data that show statistically significant differences for body weight were, time of diets in week (β = −2.05, % CI 95 −3.67 to −0.43, *p* = 0.013), for the outcome DBP, the moderator calories consumed (β = 0.0091, % CI 95 −0.0001 to 0.0013, *p* = 0.036), for the outcome glucose, the moderator calories consumed (β = 0.0031, % CI 95 −0.0021 to 0.0039, *p* = 0.001), for the outcome HOMA-IR, the moderator calories consumed (β = 0.0055, % CI 95 −0.0009 to 0.0032, *p* < 0.0001), for the outcome LDL, the moderator of age of the subjects, (β = −0.8, % CI 95 −0.16 to 0.01, *p* = 0.015), for the outcome HDL, number of frequencies per week, (β = 1.39, % CI 95 −0.36 to 2.41, *p* = 0.008) (Table 4).

## 6. Sensitivity Analysis

For the exclusion analysis to estimate the effect without the study by Esposito et al., which we believe could have had some type of influence on the results of some outcomes, for the outcome BMI by eliminating Esposito et al., 2004 [2], there are no changes in the statistical value, so for this outcome this study would not influence the results (Supplementary Figure S1), for the outcome waist circumference, we have been able to show that Esposito et al., 2004 [2], does exert a statistical effect since by including it the *p* value = 0.0001, while by excluding it’s no longer statistically significant *p* = 0.10, so in the improvement of this outcome there is an effect attributable only to one study (Supplementary Figure S2). For the DBP outcome, we have been able to show that the article by Esposito et al., 2004 [2], did not influence the results since when including it, no statistically significant differences were found between the groups; when removing this study, the results remained the same (Supplementary Figure S3). Finally, regarding the glucose outcome, when Esposito et al., 2004 [2], was eliminated, statistically significant differences were found in favor of the MD, so this study does not influence the results of this outcome (Supplementary Figure S4).

## 7. Discussion

This study aimed to evaluate the efficacy of MD compared to other therapeutic modalities or diets for the management of MS. The main findings show that MD offers significant benefits in various metabolic markers compared to standard diets or other types of diets. When combined with exercise, we believe this could increase the quality of life of these patients.

MS is a complex condition that includes various metabolic disorders such as insulin resistance, dyslipidemia, obesity, and hypertension. These disorders increase the risk of developing cardiovascular disease and type 2 diabetes, making their management a significant challenge in clinical practice. The MD has gained recognition as an effective strategy for managing MS thanks to its positive effects on various metabolic parameters.

Regarding the results of this meta-analysis, the scientific evidence showed that the MD group presented favorable outcomes in patients with MS. To analyze these results, it should be noted that this syndrome encompasses various metabolic disorders such as insulin resistance, dyslipidemia, obesity, and hypertension, which were evaluated in this study but could be analyzed separately and more comprehensively. The analysis of pooled mean differences demonstrated a significant decrease in the MD group’s insulin and blood glucose levels, with results favoring the MD. This suggests that the MD could be clinically beneficial for managing patients with MS.

Regarding previous studies that studied the effect of the MD on patients with MS, we found five previous reviews. First, the study by Kastorini et al., 2011 [24], which aimed to evaluate the effect of the MD on MS and its components, showing results showed that adherence to the MD significantly reduces the risk of MS and has a favorable impact on its components: it decreases waist circumference (−0.42 cm), triglycerides (−6.14 mg/dL), blood glucose (−3.89 mg/dL), systolic and diastolic blood pressure (−2.35 mmHg and −1.58 mmHg, respectively), and increases HDL cholesterol (+1.17 mg/dL). This work concludes that the MD is an effective and accessible intervention with a significant impact on the prevention and treatment of MS, positioning it as a key public health tool for reducing the risk of metabolic and cardiovascular diseases globally. Although this study encompasses a large number of studies, it reports results descriptively for the outcomes reported. Therefore, its results could be overestimated and underestimated. Hence, as in this study, statistical analysis using forest plots is necessary.

Godos et al., 2017 [25] examined the association between adherence to the MD and the risk of developing MS through a meta-analysis of observational studies. A total of 12 studies were included (8 cross-sectional studies and four prospective studies), with a sample of 33,847 individuals and 6342 MetS cases. The main results demonstrated that high adherence to the MD is significantly associated with a reduced risk of MS (RR: 0.81, 95% CI: 0.71–0.92). Among the MetS components, adherence to this diet significantly reduced waist circumference, blood pressure, and low HDL cholesterol levels. At the same time, no consistent associations were found with blood glucose and triglycerides. In conclusion, this meta-analysis highlights that adopting the MD can be regarded as an effective approach for the primary prevention of MS, offering significant benefits in several key components and highlighting its role in global public health. Finally, clinical trials were not included regarding the conduct and evaluation of individual outcomes, but our study did address those mentioned above.

The study by Bakaloudi et al., published in 2021 [26], evaluated the effect of MD adherence on MS components using a meta-analysis of observational studies. The results indicated that a strong adherence to the MD correlates with notable enhancements in various MetS parameters. Reduction in waist circumference (mean difference: −0.20, 95% CI: −0.40 to −0.01) and triglycerides (mean difference: −0.27, 95% CI: −0.44 to −0.11), as well as an increase in HDL cholesterol levels (mean difference: 0.28, 95% CI: 0.07 to 0.50). Nonetheless, no notable differences in blood glucose levels or systolic blood pressure were detected between groups with low and high adherence. The study concludes that the MD has a positive impact on the components of MS, although it is suggested that future research will reinforce these findings due to the high heterogeneity of the included studies. As in the previous study, we exhaustively detail the outcomes associated with the presence of MS.

The Esposito et al., 2015 [27] study evaluated the MD’s effectiveness in managing type 2 diabetes and prediabetic conditions through a meta-analysis and systematic review. Eight meta-analyses and five randomized controlled trials (RCTs) were included, with strict inclusion criteria such as a minimum duration of 6 months and at least 30 participants per group. The results showed that the MD significantly improves glycemic control (HbA1c reduction between 0.30% and 0.47%), reduces cardiovascular risk factors, such as body weight and total cholesterol, and increases HDL, compared with low-fat diets. Furthermore, a 49% higher likelihood of MS remission and a 19% to 23% reduction in the risk of developing future diabetes were observed with greater adherence to this diet. In conclusion, the study highlights that the MD is an effective and viable intervention for the comprehensive management of type 2 diabetes, supporting its use as a key tool in the prevention and treatment of metabolic and cardiovascular diseases. Although this study analyzed some components of the MS, it did not exhaustively analyze all the components of the MS.

The 2020 study by Papadaki et al. [28] evaluated the effects of the MD on metabolic health, specifically on the incidence of MS, its components, and other risk factors. Fifty-seven controlled trials with a total of 36,983 participants were included. The meta-analysis findings showed that the MD had significant beneficial effects on 18 of the 28 components evaluated, including reductions in body weight, body mass index, waist circumference, blood pressure (systolic and diastolic), glucose, insulin, total cholesterol, and triglycerides, in addition to improvements in biomarkers of inflammation and endothelial function. A reduction in the risk of cardiovascular disease (39%) and stroke (33%) was also observed, although there were no consistent effects on the use of pharmacological treatments for MS and its comorbidities. In summary, the study validates the MD as an effective approach to improving metabolic health, emphasizing its widespread benefits and advocating for its adoption in adults to help mitigate the risk of metabolic and cardiovascular diseases. The results of this meta-analysis show that since MS is a condition with multiple symptoms, the improvement outcomes must be varied, which is why grouping studies that dynamically evaluate the condition can help to have results that better cover all the aspects. This means that the study provides relevant statistical data and allows these to have a more comprehensive clinical application, which this manuscript offers.

All outcomes evaluated in this study improved with the use of the MD. As this is a multifactorial and multi-symptomatic syndrome, it is highly beneficial to have treatments that enhance clinical components that can contribute to these patients’ quality of life and health. Therefore, elements such as hypertension, glucose levels, and lipid profiles will be better for these patients. Arterial hypertension, another key criterion for the diagnosis of MS, also showed improvements in the group that followed the MD [29]. A reduction in systolic and diastolic blood pressure was observed. One of the organs responsible for this pressure regulation is the endothelium, which is responsible for vasoconstriction and vasodilation through the synthesis of molecules such as nitric oxide (NO) and endothelin-1 (ET-1). Therefore, reduced NO and increased ET-1 are associated with the pathogenesis of arterial hypertension. However, a study revealed that the MD associated with the consumption of nuts, in addition to decreasing the levels of ET-1, managed to reduce the expression of two receptors where ET-1 acts: the endothelin A receptor in the smooth muscle and the endothelin B receptor in endothelial cells, both associated with G proteins. On the other hand, the consumption of olive oil is inversely proportional to blood pressure levels, having an antihypertensive effect thanks to oleic acid, which in this study was statistically significantly more beneficial in patients treated with a MD [30,31,32,33].

In the MD group, a decline was noted in obesity-related factors such as body mass index (BMI), waist circumference, body weight, and total fat, corresponding to the reduction in the metabolic factors discussed earlier. These changes not only reflect improved weight control but also contribute to reducing the risk of obesity-related diseases, such as cardiovascular disease and fatty liver disease. The assessed factors that form the diagnostic criteria for MS demonstrated improvements in the group receiving MD treatment. This fact confirms the diet’s effectiveness and highlights its potential to guarantee health and improve patients’ quality of life. It could also be a contributing protective factor [34,35,36,37]. Therefore, understanding the beneficial effects of the foods included in this diet could improve more in-depth studies of the impact attributable to the MD. Finally, when Esposito’s study was eliminated, the results were similar and we were able to show that although this study had greater statistical weight, it did not influence the results of the forest plots that were analyzed in this study.

Our study, like many reviews on the subject, has limitations, these are mainly methodological limitations, such as having lost relevant articles, the high heterogeneity in some outcomes, we have other limitations, such as inconsistent dietary definitions which could be relevant due to the additional or minor contributions that some diets could provide in the improvement or changes in clinical parameters of MS, another factor that could influence the results is when the MD was part of a combined treatment, what we have been able to review is that in the studies included as in the meta-analysis the diet was the main treatment, but we understand that a combined diet or another type of additional therapy could increase the effect of the MD. This meta-analysis followed a rigorous and transparent methodology, in accordance with the PRISMA guidelines, the Cochrane Collaboration Handbook and the GRADE approach to assess the quality of evidence. Although the quality of the evidence ranged from low to very low, we have been transparent in reporting this since this does not depend on our review, but rather on previous studies. Given the above, although we have carried out a huge number of analyses, we believe that our results should be taken with caution.

Regarding the novelty of this study, although the effects of the Mediterranean diet on metabolic syndrome have been previously studied and we have included similar studies in the present review, this study supports its novelty and solidity, since it is statistically we have included multiple analyses that could have influenced the interpretation of previous studies, the forest plot analysis was performed on the 13 outcomes that directly influence the clinical parameters being able to see the difference in means grouped by studies, also analyzing with randomized effect the studies that had heterogeneity greater than 50%, in order to have a real effect and not influenced in a fixed effect by high heterogeneity, on the other hand, when finding studies that could have statistically influenced by their high weight, we have performed a sensitivity analysis to estimate the effect of said study on the results, which was reported in results, finally, as for each outcome analyzed, different moderators of age, time and dose of diet could have influenced the results, we have performed a meta-regression analysis to be able to analyze which factors could statistically influence In the results, finally, to evaluate the methodological quality of the included studies, we performed the Cochrane bias assessment and an analysis of each outcome with GRADE. All the above represents that the data provided in this review have undergone high-quality analyses that estimate and measure multiple variables that could influence real clinical effects.

## 8. Limitations of the Study

First, studies with conflicting results published in the literature outside the selected databases may have been omitted. Second, the search methodologies employed may have had limitations in sensitivity and specificity, which could have influenced the inclusion of specific studies. Regarding the results, some studies had to be excluded due to a lack of equivalence in the measurement units of the parameters evaluated, which prevented their inclusion in the analysis. This represents a potential bias, as the excluded data could have altered the results obtained. Finally, the high heterogeneity of some outcome comparisons between studies, which evidences data disparity, was recognized as a limitation. Recognizing this is a limitation, we analyzed this through a meta-regression of different moderators that could have influenced the heterogeneity to improve interpretability and reduce this limitation.

## 9. Conclusions

The benefits of MD have been widely documented. However, it is essential to consider that the available studies present some heterogeneity in terms of methodologies analyzed using GRADE, study populations, and specific implementation conditions. We have demonstrated that MD influences the evolution of altered clinical parameters in patients with SD, which may contribute to improving essential parameters of their quality of life. Therefore, it is crucial to consider that MD is beneficial and that, if we also add other beneficial therapeutic factors, considerable improvements could be observed in patients with SD. Current evidence supports the adoption of this approach as an effective, safe, and sustainable tool, with no significant adverse effects reported. In relation to the meta-regression performed, the age of the subjects may influence as moderating factors, which could be attributed to the more predominant presence of comorbidities of pathologies associated with MS. Another key moderator is the treatment time, which if it is of a longer duration could have improvements in outcomes that are directly associated with MS. Finally, we believe that more studies are needed with standardization of the MD, with caloric values and proportion of micro or macronutrients that standardize the use of the MD in patients with metabolic syndrome.

## Figures and Tables

**Table 1:** Features of the Included Studies.

Author	Country	Population	
		Sample Size (*n*)	Patients Mean (*SD*)
Velázquez-López 2014 [19]	Mexico	CG: 25 EG: 24	CG: 11.4 (2.9) EG: 11.2 (2.7) Children with obesity
Asoudeh 2023 [3]	Iran	CG: 35 EG: 35	CG: 14 (1) EG: 14 (1)
Konieczna 2023 [20]	Spain	CG: 761 EG: 760	CG: 65.4 (4.9) EG: 65.1 (5.1) older adults with overweight or obesity and metabolic syndrome.
Bajerska 2018 [17]	Poland	EG: 72 EC: 72	EG: 60.5 EC:60.5
Mayneris 2014 [4]	Spain	CG: 141 EG1 (MD + VOO): 142 EG2 (MD + nuts): 141	CG: EG1 (MD + VOO): EG2 (MD + nuts): The age of each group is not specified as such, only the difference between individuals with MS 67.7 (5.8) and individuals without MS 67.5 (6.1) was considered.
Fernández 2020 [8]	Spain	EG1 (MedDiet + PA): 38 EG2 (MedDiet): 37	EG1 (MedDiet + PA): 63.3 (4.4) EG2 (MedDiet): 65.6 (4.1)
Garcia 2018 [22]	Spain	Total: 79 EG: 48 CG: 31	Total: 55.7 + −7.3 EG: 55.4 + −8.4 CG: 55.9 + −6.5
Gomez 2015 [21]	Spain	Total: 601 EG: 298 CG: 303	EG: 2.39 (1.44) CG: it does not specify
Babio 2014 [1]	Spain	Total: 5801 EG1: 1982 EG2: 1885 CG: 1934	EG1: 67.1 EG2: 66.7 CG: 67.3
Esposito 2004 [2]	Italy	Total: 180 EG: 90 CG: 90	EG: 44.3 CG: 43.5
Sureda 2016 [23]	Spain	Total: 75 EG1 (Mediterranean + extra virgin olive oil): 25	EG1: EG2: CG: 55 to 80 years. The inclusion criteria were subjects without history of CVD and either the presence of Type 2 Diabetes Mellitus (T2DM) or the presence of three or more CVD risk factors.
Fortin 2018 [18]	Canada	EG: 14 CG: 14	EG: 52.1 CG: 49.8

**EG:** Experimental group; **CG:** Control group; **MedDiet:** Mediterranean diet; **MD:** Mediterranean diet; **VOO:** virgin olive oil; **MS:** Metabolic syndrome; **CVD:** cardiovascular disease; **T2DM:** Type 2 Diabetes Mellitus.

**Table 2:** Features of the Included Studies.

Author	Intervention		Outcomes	Follow-Up	Results
	Intervention	Characteristics/Dose			
Velázquez-López 2014 [19]	CG: Standard diet EG: Mediterranean diet	CG: Standard diet group was distributed with 55–60% of carbohydrates (45–50% complex and no more than 10% refined and processed sugars), 25–30% lipids, and 15% proteins. For 16 weeks. EG: Mediterranean diet: 60% carbohydrates (50% complex and no more than 10% refined and processed sugars), 25% lipids, and 15% proteins.	Weight (Kg) Height (m) BMI (kg/m^2^) Waist (cm) Hip (cm) Ratio MAC (cm) TSF (mm) Fat Mass (Kg) Lean Mass (Kg) SBP (mmHg) DBP (mmHg) Glucose (mg/dL) TC (mg/dL) TG (mg/dL) HDLC (mg/dL) LDLC (mg/dL)	16 weeks	Weight (Kg) *p* = 0.369 Height (m) *p* = 0.001 BMI (kg/m^2^) *p* = 0.001 Waist (cm) *p* = 0.0826 Hip (cm) *p* = 0.303 Ratio *p* = 0.600 MAC (cm) *p* = 0.222 TSF (mm) *p* = 0.056 Fat Mass (Kg) *p* = 0.001 Lean Mass (Kg) *p* = 0.001 SBP (mmHg) *p* = 0.229 DBP (mmHg) *p* = 0.435 Glucose (mg/dL) *p* = 0.001 TC (mg/dL) *p* = 0.001 TG (mg/dL) *p* = 0.001 HDLC (mg/dL) *p* = 0.001 LDLC (mg/dL) *p* = 0.001
Asoudeh 2023 [3]	CG: Diet supported by dietary advice based on the food pyramid EG: Prescribed Mediterranean diet	CG: Diet supported by dietary advice based on the food pyramid. Participants in the control group were only advised verbally to follow healthy eating tips recommended according to the usual Iranian diet EG: Mediterranean Diet. Energy requirements were calculated for each participant based on their weight. Diets were planned to include 53–58% of energy from carbohydrates, 15–18% from proteins and 26–30% from fats. Participants were encouraged to increase consumption of olive oil, fruits, vegetables, sweets and red meat. Refined grains were replaced with whole grains. Exchange lists were provided, and participants were instructed on their use	Weight (kg) BMI (kg/m2) WC (cm) SBP (mmHg) DBP (mmHg) FBS (mg/dL) TG (mg/dL) TC (mg/dL) HDL (mg/dL) LDL (mg/dL) Insulin (μIU/mL) HOMA-IR	12 weeks	Weight (kg) *p* = <0.001 BMI (kg/m^2^) *p* = <0.001 WC (cm) *p* = <0.001 SBP (mmHg) *p* = <0.001 DBP (mmHg) *p* = 0.64 FBS (mg/dL) *p* = <0.001 TG (mg/dL) *p* = <0.001 TC (mg/dL) *p* = 0.05 HDL (mg/dL) *p* = <0.001 LDL (mg/dL) *p* = <0.001 Insulin (μIU/mL) *p* = 0.13 HOMA-IR *p* = 0.02
Konieczna 2023 [20]	CG: Usual care, with advice to follow an ad libitum MedDiet, but no physical activity promotion. EG:energy-reduced Mediterranean diet (MedDiet) and increased physical activity (PA)	CG: Usual Care. They were given general advice to follow ad libitum the traditional MedDiet, without PA promotions, during group sessions twice a year. EG: Energy-reduced MedDiet. In addition to 30% energy reduction, the consumption of some foods (processed meats, butter, sweetened beverages, added sugar, biscuits, and bread and other refined cereals; whole grains were promoted) was limited. Participants were encouraged to progressively increase aerobic PA, aiming for 45 min of walking per day or more or the equivalent during 6 days per week, along with strength, flexibility, and balance exercises. Participants from the intervention group received support from dietitians 3 times a month (group session, individual session, and call) in the first year, along with behavioral and motivational support strategies including self-monitoring, goal setting, and problem solving.	Total fat mass (%) Total lean mass (%) Visceral fat mass (g) Total fat mass (g) Total lean mass (g) Visceral fat mass (%) Android to gynoid fat mass. Total lean mass to total fat.	3 years	Total fat mass (%) *p* = <0.001 Total lean mass (%) *p* = <0.001 Visceral fat mass (g) *p* = <0.001 Total fat mass (g) *p* = <0.001 Total lean mass (g) *p* = <0.001 Visceral fat mass (%) *p* = 0.37 Android to gynoid fat mass. *p* = 0.1 Total lean mass to total fat. *p* = <0.001
Bajerska 2018 [17]	EG: Mediterranean diet EC: Central European Diet	EG: The diet consists of: Total Fat: Approximately 37% of total energy. Monounsaturated Fatty Acids (MUFAs): 20% of total energy. Polyunsaturated Fatty Acids (PUFAs): 9% of total energy. Saturated Fatty Acids (SFAs): 8% of total energy. Protein: 18% of total energy. Carbohydrates: 45% of total energy. Dietary Fiber (DF): The ratio of soluble to insoluble fiber is 20 to 80%. Characteristic Foods: Includes typical Mediterranean products, such as olive oil (used at every meal) and nuts (five to seven per day). EC: The diet consists of: Total Fat: Approximately 27% of total energy. Monounsaturated Fatty Acids (MUFAs): 10% of total energy. Polyunsaturated Fatty Acids (PUFAs): 9% of total energy. Saturated Fatty Acids (SFAs): 8% of total energy. Protein: 18% of total energy. Carbohydrates: 55% of total energy. Dietary Fiber (DF): The ratio of soluble to insoluble fiber is 35 to 65%. Characteristic Foods: Based on typical Central European foods, such as cereals (oats and barley), legumes (peas and beans), vegetables (roots and cruciferous), and fruits (apples and plums).	Δ Body weight (kg) Δ WC (cm) Δ FM (kg) Δ FFM (kg) Δ VF (kg) Δ GLU (mg/dL) Δ INS (μU/mL) Δ HOMA2-IR Δ T-C (mg/dL) Δ LDL-C (mg/dL) Δ HDL-C (mg/dL) Δ TG (mg/dL) Δ Hcy (μM) Δ SBP (mmHg) Δ DBP (mmHg)	16 weeks	Δ Body weight (kg) *p* = <0.001 Δ WC (cm) *p* = 0.014 Δ FM (kg) *p* = 0.020 Δ FFM (kg) *p* = 0.002 Δ VF (kg) *p* = 0.285 Δ GLU (mg/dL) *p* = 0.432 Δ INS (μU/mL) *p* = 0.397 Δ HOMA2-IR *p* = 0.456 Δ T-C (mg/dL) *p* = 0.676 Δ LDL-C (mg/dL) *p* = 0.922 Δ HDL-C (mg/dL) *p* = 0.400 Δ TG (mg/dL) *p* = 0.110 Δ Hcy (μM) *p* = 0.367 Δ SBP (mmHg) *p*= 0.938 Δ DBP (mmHg) *p* = 0.519
Mayneris 2014 [4]	CG: Low-Fat Diet. EG1 (MD + VOO): Mediterranean Diet supplemented with Virgin Olive Oil. EG2 (MD + nuts): Mediterranean Diet supplemented with Nuts.	CG: They were advised to reduce all types of fats and increase the consumption of healthy foods such as vegetables and fruits. EG1 (MD + VOO): They were provided with a 1-L bottle of extra virgin olive oil every week. They were given recommendations to increase the consumption of vegetables, fruits, legumes, fish, seafood, and white meats. They were advised to limit the intake of red and processed meats, high-fat dairy products, sweets, and sugary drinks. EG2 (MD + nuts): They were provided with 30 g of mixed nuts daily (15 g of walnuts, 7.5 g of hazelnuts, and 7.5 g of almonds). They received the same dietary recommendations as the MD + VOO group.	Weight (kg) BMI (kg/m^2^) Overweight or obese (%) C Hypertension (%) D Dyslipidemia (%) E Type 2 diabetes mellitus (%) Family history of premature CHD (%) F Current smoker (%) * MetS components (%) G Elevated WC Elevated TG Reduced HDL-C Elevated BP Elevated fasting glucose * Medications (%) Aspirin or antiplatelet drugs Antihypertensive agents Hypolipidemic agents Insulin Hypoglycemic agents * Occupation (%) Worker Unemployed or unfit Retired * Education level (%) None Primary school Secondary school University	52 weeks	peso *p* = <0.001 BMI (kg/m^2^) *p* = <0.001 Overweight or obese (%) C *p* = <0.001 Hypertension (%)D *p* = 0.854 Dyslipidemia (%)E *p* = <0.001 Type 2 diabetes mellitus (%) *p* = 0.001 Family history of premature CHD (%)F *p* = 0.805 Current smoker (%) *p* = 0.118 Elevated WC *p* = <0.001 Elevated TG *p* = <0.001 Reduced HDL-C *p* = <0.001 Elevated BP *p* = <0.001 Elevated fasting glucose *p* = <0.001 Aspirin or antiplatelet drugs *p* = 0.593 Antihypertensive agents *p* = 0.110 Hipolypidemic agents *p* = 0.001 Insulin *p* = 0.783 Hypoglycemic agents *p* = 0.001 Worker *p* = 0.078 Unemployed or unfit *p* = 0.141 Retired *p* = 0.745 None *p* = 0.237 Primary school *p* = 0.116 Secondary school *p* = 0.012 University *p* = 0.995
Fernández 2020 [8]	EG1 (er-MedDiet + PA): Mediterranean diet with caloric restriction combined with the promotion of physical activity and behavioral support. EG2 (MedDiet): Mediterranean diet without caloric restriction and received traditional healthcare.	EG1 (er-MedDiet + PA): The Energy-Restricted Mediterranean Diet combined the principles of the Mediterranean diet with caloric restriction to promote weight loss. It included high consumption of fruits, vegetables, legumes, whole grains, fish, and extra virgin olive oil, with reduced consumption of red/processed meats, added sugars, and high-fat dairy. Participants engaged in increased physical activity levels, focusing on aerobic and resistance exercises. Personalized counseling, behavioral support and group sessions were offered to improve adherence to the diet and exercise program. EG2 (MedDiet): The Mediterranean Diet without Caloric Restriction followed the traditional Mediterranean diet principles, focusing on a high intake of fruits, vegetables, legumes, whole grains, fish, and virgin olive oil, while limiting red/processed meats, added sugars, and high-fat dairy. Participants received standard healthcare with general recommendations but no specific focus on caloric restriction, structured physical activity, or behavioral support.	BMI, kg/m^2^ Serum glucose, mg/dL Serum HbA1c, % Serum HDL cholesterol, mg/dL Serum triglycerides, mg/dL	26 weeks	BMI, kg/m^2^ *p* = <0.001 Serum glucose, mg/dL *p* = 0.708 Serum HbA1c, % *p* = 0.444 Serum HDL cholesterol, mg/dL *p* = 0.205 Serum triglycerides, mg/dL *p* = 0.635
Garcia 2018 [22]	CG: Single workshop providing basic information about MetS and associated cardiovascular risks. EG: CBT	EG: Cognitive Behavioral Therapy (CBT) was delivered in 12 weekly 90-min sessions (10–12 participants) led by a psychologist. The program addressed factors related to Metabolic Syndrome (MetS) and CBT, negative beliefs about diet and exercise, problem solving for lifestyle changes, strategies to manage impulsivity, stress and anger, assertiveness training, confidence building and strategies for long-term adherence to healthy habits with support from family and professionals. CG: A 90-min workshop was conducted once for each of the four subgroups, each consisting of approximately 10 to 15 participants. It provided fundamental information on MetS and its associated cardiovascular risks, presenting standard therapeutic strategies in accordance with the Nutrition, Physical Activity, and Obesity Prevention Strategy of the Spanish Agency for Food Safety and Nutrition.	IMC (kg/m^2^) PA (mmHg) HDLc (mg/dL) MedDiet MEDAS-14 MMSE	12 weeks	Waist circumference *p*= 0.001 Triglycerides *p* = 0.018 Mediterranean diet *p* = 0.000
Gomez 2015 [21]	EG: Mediterranean Diet and physical activity CG: General recommendations on healthy diet and physical exercise	EG: The Mediterranean-style diet emphasized olive oil, vegetables, fruits, legumes, and fish with limited red or processed meats, dairy milk, sugary drinks, and sweets. Overweight or obese patients followed a 600 kcal/day deficit diet, aiming for a ≥5% weight loss, calculated using the Harris-Benedict equation. Daily exercise was encouraged, with a minimum of 150 min of walking per week. A total of 27 visits were conducted throughout the study, including 9 medical visits and 18 nursing visits. CG: General recommendations on heart-healthy diet and physical exercise. Participants received at least 4 medical consultations and 4 nursing visits per year.	Waist circumference (cm) Weight (kg) Normoweight (%) Overweight (%) Obesity (%) BMI (kg/m^2^) SBP (mmHg)DBP (mmHg) Low educational level (%) Sedentary lifestyle (%) Smoking (%) Glycemia (mg/dL) HbA1c (%) Creatinine (mg/dL) Uric acid (mg/dL) Total cholesterol (mg/dL) LDL cholesterol (mg/dL) HDL cholesterol (mg/dL) Triglycerides (mg/dL) Type 2 diabetes mellitus, *n* (%) Cardiovascular disease, *n* (%)	3 years	Waist circumference (cm) *p* = 0.001 Weight (kg) *p* = ns BMI (kg/m^2^) *p* = ns SBP (mmHg) *p* = 0.004 DBP (mmHg) *p* = <0.001 Glycemia (mg/dL) *p* = ns HbA1c (%) *p* = ns Total cholesterol (mg/dL) *p* = ns LDL cholesterol (mg/dL) *p* = ns HDL cholesterol (mg/dL) *p* = 0.05 Triglycerides (mg/dL) *p* = ns Metabolic syndrome, *n* (%) *p* = ns Number of components of metabolic syndrome *p* = 0.02 Antihypertensive drugs, *p* = ns Lipid-lowering drugs, *p* = ns Antidiabetic drugs *p* = ns Type 2 Diabetes mellitus *p* = ns Cardiovascular disease *p* = ns
Babio 2014 [1]	EG1: Mediterranean diet and extra-virgin olive oil EG2: Mediterranean diet and nuts CG: low-fat diet	EG1: Mediterranean diet with an emphasis on the consumption of extra virgin olive oil, 1 L per week. Participants were given sessions to provide information on the Mediterranean diet, meal plans, and recipes. EG2: Mediterranean diet with an emphasis on the consumption of a mixed nut blend (30 g/day): 15 g of walnuts, 7.5 g of hazelnuts, and 7.5 g of almonds. Participants were given sessions to provide information on the Mediterranean diet, meal plans, and recipes. CG: Diet low in both animal and vegetable fats. Participants were given sessions to receive recommendations on reducing fats in their diet.	Former smoker (%) Current smoker (%) BMI (kg/m^2^) Waist circumference (cm) Leisure time physical activity (METs/min per d) Mediterranean diet score (0–14) T2DM (%) Metabolic syndrome (%) Central obesity (%) Hypertriglyceridemia (%) Low HDL cholesterol (%) Hypertension (%) High fasting plasma glucose (%) Insulin (%) Hypoglycemic agents (%) Fibrates (%) Statins (%) Aspirin (%) Antihypertensive agents (%)	4.8 years	Former smoker *p* = 0.08 Current smoker *p* = 0.8 BMI (kg/m2) *p* = <0.001 Waist circumference (cm) *p* = 0.08 Leisure time physical activity (METs/min per d) ***p* = <0.001** Mediterranean diet score (0–14) *p* = <0.001 T2DM *p* = 0.1 Metabolic syndrome *p* = 0.07 Central obesity *p* = <0.001 Hypertriglyceridemia *p* = 0.4 Low HDL cholesterol 0.8 Hypertension *p* = 0.3 High fasting plasma glucose *p* = 0.6 Insulin *p* = 0.1 Hypoglycemic agents *p* = 0.02 Fibrates *p* = 0.3 Statins *p* = 0.2 Aspirin *p* = 0.5 Antihypertensive agents *p* = 0.3
Esposito 2004 [2]	EG: Mediterranean diet CG: macronutrient composition similar to mediterranean diet	EG: Carbohydrates 50–60%, proteins 15–20%, total fat less than 30%, saturated fat less than 10%, cholesterol consumption less than 300 mg per day. Patients were advised to consume at least 250 to 300 g of fruits, 125 to 150 g of vegetables and 25 to 50 g of walnuts per day. They were also encouraged to consume 400 g of whole grains (legumes, rice, maize and wheat) daily and to increase their consumption of olive oil. Patients had monthly sessions with the nutritionist for the first year and bi-monthly sessions for the second year. CG: Carbohydrates 50–60%, proteins 15–20%, total fat less than 30%. Patients had bi-monthly sessions with study personnel for 2 years.	No. of components of the metabolic syndrome (%) Body weight (kg) BMI (kg/m^2^) Waist circumference (cm) Plasma glucose (mg/dL) Serum insulin (μU/mL) HOMA score (%) Total cholesterol (mg/dL) HDL-C (mg/dL) Triglycerides (mg/dL) Systolic blood pressure (mmHg) Diastolic blood pressure (mmHg) Endothelial function score hs-CRP (mg/L) IL-6 (pg/mL) IL-7 (pg/mL) IL-18 (pg/mL)	2 years	No. of components of the metabolic syndrome *p* = 0.34 Body weight *p* = 0.36 BMI (kg/m^2^) *p* = 0.55 Waist circumference (cm) *p* = 0.62 Plasma glucose (mg/dL) *p* = 0.43 Serum insulin (μU/mL) *p* = 0.15 HOMA score *p* = 0.18 Total cholesterol (mg/dL) *p* = 0.18 HDL-C (mg/dL) *p* = 0.35 Triglycerides (mg/dL) *p* = 0.24 Systolic blood pressure (mmHg) *p* = 0.11 Diastolic blood pressure (mmHg) *p* = 0.21 Endothelial function score *p* = 0.13 hs-CRP (mg/L) *p* = 0.25 IL-6 (pg/mL) *p* = 0.14 IL-7 (pg/mL) *p* = 0.12 IL-18 (pg/mL) *p* = 0.1
Sureda 2016 [23]	EG1: MeDiet supplemented with extra virgin olive oil (MeDiet + EVOO), EG2: MeDiet with nuts (MeDiet + nuts) CG: Low-fat diet	EG: Participants in the MeDiet groups received free EVOO (15 L for 3 months) or mixed nuts (15 g walnuts, 7.5 g hazelnuts, 7.5 g almonds daily for 3 months) with excess provided for family needs. A 1-h group session (up to 20 participants) for each MeDiet group with a dietician was held to improve compliance, focusing on increasing the MeDiet 14-item score. Recommendations included using olive oil for cooking, increasing intake of vegetables, nuts, fish and white meat, preparing home-made sauces to dress vegetables, pasta, rice and other dishes, and moderate red wine consumption for drinkers. No energy restrictions or physical activity interventions were implemented. CG: Participants allocated to a low-fat diet were advised to reduce all types of fat and were given written recommendations according to the American Heart Association guidelines.	Superoxide dismutase activity (pkat/L) Catalase activity (k/L) Myeloperoxidase activity (μkat/L) Xanthine oxidase activity (U/L) Superoxide dismutase protein level (%) Catalase protein level (%) Myeloperoxidase protein level (%) Xanthine oxidase protein level (%) (Datos de arriba en el **plasma**) Superoxide dismutase activity (pkat/mL) Catalase Activity (k/mL) **(Red blood cells)**	5 years	Superoxide dismutase activity (pkat/L) ***p* < 0.003** Catalase activity (k/L) ***p* < 0.004** Myeloperoxidase activity (μkat/L) *p* = 0.690 Xanthine oxidase activity (U/L) ***p* = 0.008** Superoxide dismutase protein level (%) ***p* = 0.033** Catalase protein level (%) *p* = 0.823 Myeloperoxidase protein level (%)*p* = 0.241 Xanthine oxidase protein level (%) *p* = 0.296 (Datos de arriba en el **plasma**) Superoxide dismutase activity (pkat/mL) *p* = 0.233 Catalase Activity (k/mL) *p* = 0.225 **(Red blood cells)**
Fortin 2018 [18]	EG: Mediterranean diet CG: Low-fat diet	EG: included using olive oil as the main source of fat, multiple servings of fish per week and a limited intake of red and processed meat. CG: Low-fat diet included lean cuts of meat and limited fried and cholesterol-rich foods. Each group received dietary teaching sessions, once a week for the first month, bi-weekly for the second month, and then monthly.	Body weight (kg) Waist circumference (cm) BMI (kg/m^2^) Body fat (%) HbA1c (%) Estimated glucose disposal rate (eGDR) Hypoglycemia episode (*n* per day) Systolic blood pressure (mmHg) Diastolic blood pressure (mmHg) Total cholesterol (mmol/L) HDL-cholesterol (mmol/L) LDL-cholesterol (mmol/L) Triglycerides (mmol/L) Apolipoprotein-B (g/L) C-reactive protein (mg/L)	6 meses	Body weight (kg) *p* = 0.96 Waist circumference (cm) *p* = 0.93 BMI (kg/m^2^) *p* = 0.24 Body fat *p* = 0.27 HbA1c *p* = 0.7 Estimated glucose disposal rate (eGDR) *p* = 0.19 Hypoglycemia episode (*n* per day) *p* = 85 Systolic blood pressure (mmHg) *p* = 0.21 Diastolic blood pressure (mmHg) *p* = 0.76 Total cholesterol (mmol/L) *p* = 0.61 HDL-cholesterol (mmol/L) *p* = 0.1 LDL-cholesterol (mmol/L) *p* = 0.89 Triglycerides (mmol/L) *p* = 0.61 Apolipoprotein-B (g/L) *p* = 0.1 C-reactive protein (mg/L) *p* = 0.84

**EG**: Experimental group; **SBP**: Systolic blood pressure; **MAC**: Mid-arm circumference; **TC**: Total cholesterol; **CG**: Control group; **BMI**: Body Mass Index; **TSF**: Tricipital skinfold; **DBP**: Diastolic blood pressure. **TG**: triglycerides; **HDLC**: low high-density lipoprotein cholesterol; **LDLC**: Low density lipoprotein cholesterol; **WC**: Waist circumference; **HOMA-IR**: Homeostatic Model Assessment of Insulin Resistance; **FBS**: fasting blood sugar; **HDL**: high-density lipoprotein-cholesterol, **LDL**: low-density lipoprotein-cholesterol, **MedDiet**: Mediterranean diet; **PA**: physical activity; **HbA1c**:glycosylated hemoglobin; **PUFAs**: Polyunsaturated Fatty Acids, **SFAs**: Saturated Fatty Acids; **DF**: dietary fiber; **MUFAs**: Monounsaturated Fatty Acids; **FM**: fat mass; **FFM**: fat-free mass; **VF**: visceral fat; **GLU**: glucose; **INS**: insulin; **HOMA2-IR**: Homeostatic Model Assessment of Insulin Resistance; **T-C**: Total cholesterol; **TG;** Hcy: homocysteine; **MD**: Mediterranean diet; **VOO**: virgin olive oil; **CHD**: coronary heart disease; **MetS**: Metabolic syndrome; **MS**: Metabolic syndrome; **CBT**: Cognitive Behavioral Therapy; **MEDAS-14**: Med-Diet adherence screener; **MMSE**: Mini-Mental State Examination; **HOMA**: homeostasis model assessment; **hs-CRP**: high-sensitivity C-reactive protein; **IL-6**: interleukin 6; **IL-7**: interleukin 7; **IL-18**: interleukin 18; **CVD**: cardiovascular disease; **T2DM**: Type 2 Diabetes Mellitus; **EVOO**: extra-virgin olive oil; **eGDR**: Estimated glucose disposal rate. * means those parameters have sub-parameters below.

**Table 3:** Characteristics included studies in this systematic review.

Parameter	Normal Reference Values	Reference Values in Diabetics	Values Reported by the Articles (Average)	Reference Values for Metabolic Syndrome (Expected to Increase)	Additional Considerations
**Body weight**	It depends on weight and height	It depends on weight and height	77	It depends on weight and height	No present
***BMI (IMC)***	18.5–24.9 kg/m^2^	18.5–24.9 kg/m^2^	28.4 kg/m^2^	>30 kg/m^2^	No present
**Waist circumference**	<88 cm (women) <102 cm (males)	<88 cm (women) <102 cm (males)	97 cm	>90 cm (women) >102 cm (males)	No present
**SBP**	<120 mmHg	130–139 mmHg	122 mmHg	>130 mmHg	No present
**DBP**	<80 mmHg	80–85 mmHg	76 mmHg	>85 mmH	No present
**Triglycerides**	<150 mg/dL	<135 mg/dL	162 mg/dL	>150 mg/dL	The stated goals apply to diabetic patients without coronary heart disease.
**Glucose**	<100 mg/dL	<140 mg/dL	110 mg/dL	>110 mg/dL	Goals vary depending on the person’s condition, age, life expectancy, and presence of other comorbidities.
**Total cholesterol**	<200 mg/dL	>240 mg/dL	186.53 mg/dL	>240 mg/dL	The stated goals apply to diabetic patients without coronary heart disease.
**HDL**	>50 mg/dL (women) >40 mg/dL (men)	<50 mg/dL (women) <40 mg/dL (men)	48.45 mg/dL	<50 mg/ dL (women) <40 mg/dL (men)	The stated goals apply to diabetic patients without coronary heart disease.
**LDL**	<100	>130 mg/dL	107.43 mg/dL	>130 mg/dL	The goal varies depending on whether the person has a history of cardiovascular disease (CVD).
**Body fat**	10–20% (men) 20–30% (women)	There are no official reference values; these vary depending on the person and their health status.	34.03 kg	>25% (men) >35% (women)	Reference values should be interpreted in the context of overall health, physical activity level, and other indicators such as body mass index (BMI) and age.
**HOMA-IR**	<2.5	>2.5	2.98	>2.5	It may vary depending on each case
**Insulin**	2–25 µU/ mL	Insulin levels may be elevated, especially if you are in a phase of insulin resistance	0.0894 mg/dL	>25 µU/mL	Glucose reference values vary depending on factors such as physiological status (pregnancy or not), time elapsed since last food intake, and individual patient characteristics.

**Table 4:** Meta-Regression.

Outcome	Moderator	B	95% LLCI	95% ULCI	*p*-Value
Body weight	Age	−0.04	−0.17	0.09	0.55077
Body weight	Calories	0.00	0.00	0.00	0.50502
Body weight	Diet weeks	−2.05	−3.67	−0.43	0.01316
Body weight	Frequency per week	−0.08	−0.62	0.47	0.78602
BMI	Age	0.01	0.00	0.01	0.07544
BMI	Calories	0.00	0.00	0.00	0.09745
BMI	Diet weeks	0.02	0.00	0.05	0.10371
BMI	Frequency per week	0.14	−0.15	0.42	0.34744
Cholesterol	Age	0.00	−0.66	0.67	0.98971
Cholesterol	Calories	0.00	−0.02	0.01	0.73085
Cholesterol	Diet weeks	−0.74	−2.85	1.38	0.49607
Cholesterol	Frequency per week	6.34	−4.06	16.75	0.23222
DBP	Age	−0.03	−0.12	0.05	0.44586
DBP	Calories	0.00	0.00	0.00	0.03633
DBP	Diet weeks	−0.15	−0.39	0.10	0.24880
DBP	Frequency per week	−0.75	−3.01	1.51	0.51456
Glucose	Age	0.03	−0.23	0.29	0.83103
Glucose	Calories	0.00	0.00	0.00	0.00179
Glucose	Diet weeks	−0.37	−1.24	0.50	0.40457
Glucose	Frequency per week	0.94	−3.66	5.55	0.68765
HOMA−IR	Age	0.00	−0.05	0.05	0.96735
HOMA−IR	Calories	0.00	0.00	0.00	0.00001
HOMA−IR	Diet weeks	−0.05	−0.19	0.10	0.51892
Insulin	Age	−0.03	−0.13	0.08	0.62624
Insulin	Calories	0.00	0.00	0.00	0.28858
Insulin	Diet weeks	0.01	−0.38	0.40	0.95117
LDL	Age	0.42	0.08	0.76	0.01563
LDL	Calories	−0.01	−0.03	0.02	0.60745
LDL	Diet weeks	0.94	−1.63	3.51	0.47353
LDL	Frequency per week	2.64	−10.50	15.78	0.69337
HDL	Age	−0.08	−0.16	0.01	0.08473
HDL	Calories	0.00	0.00	0.00	0.92310
HDL	Diet weeks	−0.20	−0.50	0.10	0.18456
HDL	Frequency per week	1.39	0.36	2.41	0.00834
Triglycerides	Age	0.70	−0.33	1.72	0.18370
Triglycerides	Calories	−0.01	−0.04	0.02	0.57344
Triglycerides	Diet weeks	0.80	−4.68	6.28	0.77523
Triglycerides	Frequency per week	20.52	−8.20	49.24	0.16132
SBP	Age	−0.01	−0.20	0.18	0.92662
SBP	Calories	0.00	0.00	0.00	0.39526
SBP	Diet weeks	0.01	−0.75	0.77	0.97900
SBP	Frequency per week	−2.11	−5.13	0.91	0.17148
Waist circumference	Age	0.03	−0.04	0.11	0.36890
Waist circumference	Calories	0.00	0.00	0.00	0.30175
Waist circumference	Diet weeks	0.01	−0.30	0.32	0.94285
Waist circumference	Frequency per week	0.47	−1.43	2.36	0.62964

## Supplementary Materials

The following supporting information can be downloaded at https://www.mdpi.com/article/10.3390/ijms26125887/s1.

## References

[1] Babio N., Toledo E., Estruch R., Ros E., Martínez-González M.A., Castañer O., Bulló M., Corella D., Arós F., Gómez-Gracia E. (2014). Mediterranean diets and metabolic syndrome status in the PREDIMEDrandomized trial. CMAJ.

[2] Esposito K., Marfella R., Ciotola M., Di Palo C., Giugliano F., Giugliano G., D’Armiento M., D’Andrea F., Giugliano D. (2004). Effect of a mediterranean-style diet on endothelial dysfunction and markers of vascular inflammation in the metabolic syndrome: A randomized trial. JAMA.

[3] Asoudeh F., Fallah M., Aminianfar A., Djafarian K., Shirzad N., Clark C.C.T., Larijani B., Esmaillzadeh A. (2023). The effect of Mediterranean diet on inflammatory biomarkers and components of metabolic syndrome in adolescent girls. J. Endocrinol. Investig..

[4] Mayneris-Perxachs J., Sala-Vila A., Chisaguano M., Castellote A.I., Estruch R., Covas M.I., Fitó M., Salas-Salvadó J., Martínez-González M.A., Lamuela-Raventós R. (2014). Effects of 1-year intervention with a Mediterranean diet on plasma fatty acid composition and metabolic syndrome in a population at high cardiovascular risk. PLoS ONE.

[5] Sofi F., Dinu M., Pagliai G., Cesari F., Marcucci R., Casini A. (2016). Mediterranean versus vegetarian diet for cardiovascular disease prevention (the CARDIVEG study): Study protocol for a randomized controlled trial. Trials.

[6] Garcia M., Bihuniak J.D., Shook J., Kenny A., Kerstetter J., Huedo-Medina T.B. (2016). The Effect of the Traditional Mediterranean-Style Diet on Metabolic Risk Factors: A Meta-Analysis. Nutrients.

[7] Fernández J.M., Rosado-Álvarez D., Da Silva Grigoletto M.E., Rangel-Zúñiga O.A., Landaeta-Díaz L.L., Caballero-Villarraso J., López-Miranda J., Pérez-Jiménez F., Fuentes-Jiménez F. (2012). Moderate-to-high-intensity training and a hypocaloric Mediterranean diet enhance endothelial progenitor cells and fitness in subjects with the metabolic syndrome. Clin. Sci..

[8] Fernández-García J.C., Martínez-Sánchez M.A., Bernal-López M.R., Muñoz-Garach A., Martínez-González M.A., Fitó M., Salas-Salvadó J., Tinahones F.J., Ramos-Molina B. (2020). Effect of a lifestyle intervention program with energy-restricted Mediterranean diet and exercise on the serum polyamine metabolome in individuals at high cardiovascular disease risk: A randomized clinical trial. Am. J. Clin. Nutr..

[9] Salas-Salvadó J., Fernández-Ballart J., Ros E., Martínez-González M.A., Fitó M., Estruch R., Corella D., Fiol M., Gómez-Gracia E., Arós F. (2008). Effect of a Mediterranean diet supplemented with nuts on metabolic syndrome status: One-year results of the PREDIMED randomized trial. Arch. Intern. Med..

[10] Page M.J., McKenzie J.E., Bossuyt P.M., Boutron I., Hoffmann T.C., Mulrow C.D., Shamseer L., Tetzlaff J.M., Akl E.A., Brennan S.E. (2021). The PRISMA 2020 statement: An updated guideline for reporting systematic reviews. BMJ.

[11] Rochlani Y., Pothineni N.V., Kovelamudi S., Mehta J.L. (2017). Metabolic syndrome: Pathophysiology, management, and modulation by natural compounds. Ther. Adv. Cardiovasc. Dis..

[12] Ghoshal U.C., Sonthalia N., Roy A., Goenka M.K. (2025). Metabolic Syndrome and Gastroesophageal Reflux Disease: Clinical Remission With Treatment, Beyond an Epidemiological Association. J. Neurogastroenterol. Motil..

[13] Thapa K., Khan H., Chahuan S., Dhankhar S., Kaur A., Garg N., Saini M., Singh T.G. (2025). Insights into therapeutic approaches for the treatment of neurodegenerative diseases targeting metabolic syndrome. Mol. Biol. Rep..

[14] Chavez M., Ramirez A., Hernández-Vásquez A., Comandé D., Azañedo D. (2025). Impact of subgingival periodontal treatment on systemic markers of inflammation in patients with metabolic syndrome: A systematic review of randomized clinical trials. Front. Oral Health.

[15] Barcot O., Ivanda M., Buljan I., Pieper D., Puljak L. (2021). Enhanced access to recommendations from the Cochrane Handbook for improving authors’ judgments about risk of bias: A randomized controlled trial. Res. Synth. Methods.

[16] Guyatt G., Oxman A.D., Akl E.A., Kunz R., Vist G., Brozek J., Norris S., Falck-Ytter Y., Glasziou P., DeBeer H. (2011). GRADE guidelines: 1. Introduction-GRADE evidence profiles and summary of findings tables. J. Clin. Epidemiol..

[17] Bajerska J., Chmurzynska A., Muzsik A., Krzyżanowska P., Mądry E., Malinowska A.M., Walkowiak J. (2018). Weight loss and metabolic health effects from energy-restricted Mediterranean and Central-European diets in postmenopausal women: A randomized controlled trial. Sci. Rep..

[18] Fortin A., Rabasa-Lhoret R., Lemieux S., Labonté M.E., Gingras V. (2018). Comparison of a Mediterranean to a low-fat diet intervention in adults with type 1 diabetes and metabolic syndrome: A 6-month randomized trial. Nutr. Metab. Cardiovasc. Dis..

[19] Velázquez-López L., Santiago-Díaz G., Nava-Hernández J., Muñoz-Torres A.V., Medina-Bravo P., Torres-Tamayo M. (2014). Mediterranean-style diet reduces metabolic syndrome components in obese children and adolescents with obesity. BMC Pediatr..

[20] Konieczna J., Ruiz-Canela M., Galmes-Panades A.M., Abete I., Babio N., Fiol M., Martín-Sánchez V., Estruch R., Vidal J., Buil-Cosiales P. (2023). An Energy-Reduced Mediterranean Diet, Physical Activity, and Body Composition: An Interim Subgroup Analysis of the PREDIMED-Plus Randomized Clinical Trial. JAMA Netw. Open.

[21] Gomez-Huelgas R., Jansen-Chaparro S., Baca-Osorio A.J., Mancera-Romero J., Tinahones F.J., Bernal-López M.R. (2015). Effects of a long-term lifestyle intervention program with Mediterranean diet and exercise for the management of patients with metabolic syndrome in a primary care setting. Eur. J. Intern. Med..

[22] Garcia-Silva J., NNavarrete N., Peralta-Ramírez M.I., García-Sánchez A., Ferrer-González M.Á., Caballo V.E. (2018). Efficacy of Cognitive Behavioral Therapy in Adherence to the Mediterranean Diet in Metabolic Syndrome Patients: A Randomized Controlled Trial. J. Nutr. Educ. Behav..

[23] Sureda A., Bibiloni M.D., Martorell M., Buil-Cosiales P., Marti A., Pons A., Tur J.A., Martinez-Gonzalez M.Á., PREDIMED Study Investigators (2016). Mediterranean diets supplemented with virgin olive oil and nuts enhance plasmatic antioxidant capabilities and decrease xanthine oxidase activity in people with metabolic syndrome: The PREDIMED study. Mol. Nutr. Food Res..

[24] Kastorini C.M., Milionis H.J., Esposito K., Giugliano D., Goudevenos J.A., Panagiotakos D.B. (2011). The effect of Mediterranean diet on metabolic syndrome and its components: A meta-analysis of 50 studies and 534,906 individuals. J. Am. Coll. Cardiol..

[25] Godos J., Zappalà G., Bernardini S., Giambini I., Bes-Rastrollo M., Martinez-Gonzalez M. (2017). Adherence to the Mediterranean diet is inversely associated with metabolic syndrome occurrence: A meta-analysis of observational studies. Int. J. Food Sci. Nutr..

[26] Bakaloudi D.R., Chrysoula L., Kotzakioulafi E., Theodoridis X., Chourdakis M. (2021). Impact of the Level of Adherence to Mediterranean Diet on the Parameters of Metabolic Syndrome: A Systematic Review and Meta-Analysis of Observational Studies. Nutrients.

[27] Esposito K., Maiorino M.I., Bellastella G., Chiodini P., Panagiotakos D., Giugliano D. (2015). A journey into a Mediterranean diet and type 2 diabetes: A systematic review with meta-analyses. BMJ Open.

[28] Papadaki A., Nolen-Doerr E., Mantzoros C.S. (2020). The Effect of the Mediterranean Diet on Metabolic Health: A Systematic Review and Meta-Analysis of Controlled Trials in Adults. Nutrients.

[29] Pasanisi P., Gargano G., Gaetana Di Mauro M., Cortellini M., Casagrande A., Villarini A., Bruno E., Roveda E., Saibene G., Venturelli E. (2018). A randomized controlled trial of Mediterranean diet and metformin to prevent age-related diseases in people with metabolic syndrome. Tumori J..

[30] Tuttolomondo A., Simonetta I., Daidone M., Mogavero A., Ortello A., Pinto A. (2019). Metabolic and Vascular Effect of the Mediterranean Diet. Int. J. Mol. Sci..

[31] Daidone M., Di Chiara T., Del Cuore A., Casuccio A., Salamone G., Di Raimondo D., Tuttolomondo A. (2025). Mediterranean diet and hypertension: Relationship between adherence to a Mediterranean diet and arterial hypertension. BMC Nutr..

[32] Scaglione S., Di Chiara T., Daidone M., Tuttolomondo A. (2025). Effects of the Mediterranean Diet on the Components of Metabolic Syndrome Concerning the Cardiometabolic Risk. Nutrients.

[33] Kim J.H., Lee J.W., Lee Y., Nam C.M., Kwon Y.J. (2025). Impact of Mediterranean Diet Adherence on the Incidence of New-Onset Hypertension in Adults With Obesity in Korea: A Nationwide Cohort Study. J. Clin. Hypertens..

[34] Micó-Pérez R.M., Hernández Segura N., Martín-Sánchez V., Barquilla-García A., Velilla-Zancada S.M., Polo-García J., Prieto-Díaz M.Á., Pallares-Carratala V., Segura-Fragoso A., Ginel-Mendoza L. (2025). Physical activity and metabolic syndrome in primary care patients in Spain. PLoS ONE.

[35] Tárraga Marcos P.J., López-González Á.A., Martínez-Almoyna Rifá E., Paublini Oliveira H., Martorell Sánchez C., Tárraga López P.J., Ramírez-Manent J.I. (2025). The Prevalence of Metabolic Syndrome and Hypertriglyceridemic Waist Based on Sociodemographic Variables and Healthy Habits in Healthcare Workers: A Retrospective Study. Life.

[36] Cornali K., Di Lauro M., Marrone G., Masci C., Montalto G., Giovannelli A., Schievano C., Tesauro M., Pieri M., Bernardini S. (2025). The Effects of a Food Supplement, Based on Co-Micronized Palmitoylethanolamide (PEA)-Rutin and Hydroxytyrosol, in Metabolic Syndrome Patients: Preliminary Results. Nutrients.

[37] Pranjić I., Sila S., Lulić Kujundžić S., Dodig M., Vestergaard Larsen A., Kranjčec I. (2025). Metabolic Sequelae and Quality of Life in Early Post-Treatment Period in Adolescents with Hodgkin Lymphoma. J. Clin. Med..

