# Supplementary material for: Comparison of the Mediterranean Diet and Other Therapeutic Strategies in Metabolic Syndrome: A Systematic Review and Meta-Analysis
Source: Int J Mol Sci. 2025 Jun 19;26(12):5887. doi: 10.3390/ijms26125887 (PMC12192665; doi:10.3390/ijms26125887)

Supplementary Figure S1. BMI without Esposito et al., 2004.

Study or Subgroup	Mediterranean diet Mean	SD	Total	Standard diet Mean	SD	Total	Weight	Mean Difference IV, Fixed, 95% CI
Asoudeh 2023	-1.1	0.63	35	-0.1	0.63	35	75.5%	-1.00 [-1.30, -0.70]
Fortin 2018	-0.7	2.85	12	-1.1	3.65	14	1.1%	0.40 [-2.10, 2.90]
Velasquez 2014	-1.1	0.87	24	0.1	1.02	25	23.4%	-1.20 [-1.73, -0.67]
**Total (95% CI)**			**71**			**74**	**100.0%**	**-1.03 [-1.29, -0.78]**

Heterogeneity: $Chi^2 = 1.69$, df = 2 (P = 0.43); $I^2 = 0\%$
Test for overall effect: Z = 7.89 (P < 0.00001)

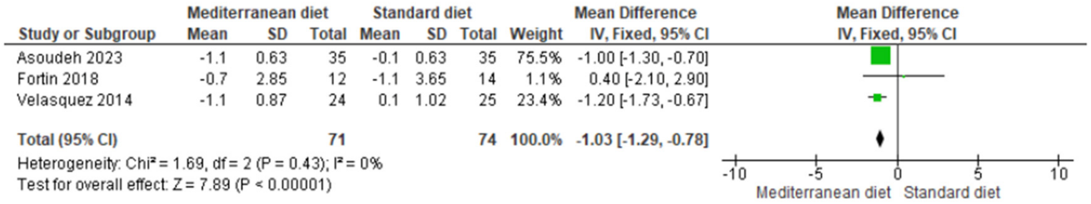

Supplementary Figure S2. waist circumference without Esposito et al., 2004

Study or Subgroup	Mediterranean diet Mean	SD	Total	Standard diet Mean	SD	Total	Weight	Mean Difference IV, Random, 95% CI
Asoudeh 2023	-2.9	1.46	35	-0.3	1.46	35	39.5%	-2.60 [-3.28, -1.92]
Bajerska 2018	-7.4	3.55	67	-7.5	4.05	63	34.9%	0.10 [-1.21, 1.41]
Fortin 2018	-3.5	9.6	12	-1.5	8.65	14	6.4%	-2.00 [-9.07, 5.07]
Velasquez 2014	-0.2	5.62	24	2.5	6	25	19.2%	-2.70 [-5.95, 0.55]
**Total (95% CI)**			**138**			**137**	**100.0%**	**-1.64 [-3.58, 0.30]**

Heterogeneity: $Tau^2 = 2.36$; $Chi^2 = 12.94$, df = 3 (P = 0.005); $I^2 = 77\%$
Test for overall effect: Z = 1.65 (P = 0.10)

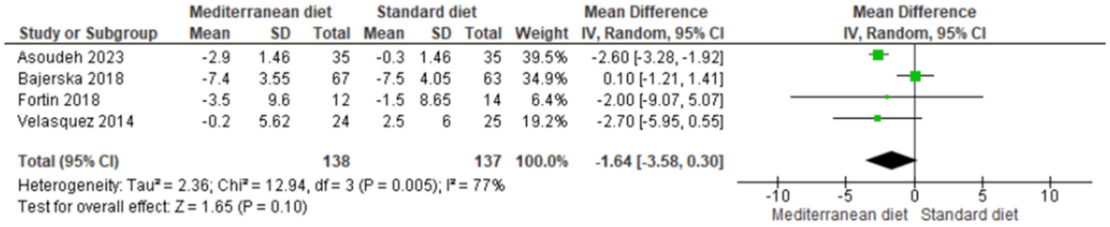

Supplementary Figure S3. Diastolic blood presion without Esposito et al., 2004

Study or Subgroup	Mediterranean diet Mean	SD	Total	Standard diet Mean	SD	Total	Weight	Mean Difference IV, Fixed, 95% CI
Asoudeh 2023	0.012	1.05	35	0	1.05	35	96.2%	0.01 [-0.48, 0.50]
Bajerska 2018	-6.7	9.8	67	-8.1	8.1	63	2.4%	1.40 [-1.68, 4.48]
Fortin 2018	-2	9.3	12	-2	7	14	0.6%	0.00 [-6.41, 6.41]
Velasquez 2014	1.7	10.75	24	1.5	7.91	25	0.8%	0.20 [-5.10, 5.50]
**Total (95% CI)**			**138**			**137**	**100.0%**	**0.05 [-0.43, 0.53]**

Heterogeneity: $Chi^2 = 0.76$, df = 3 (P = 0.86); $I^2 = 0\%$
Test for overall effect: Z = 0.19 (P = 0.85)

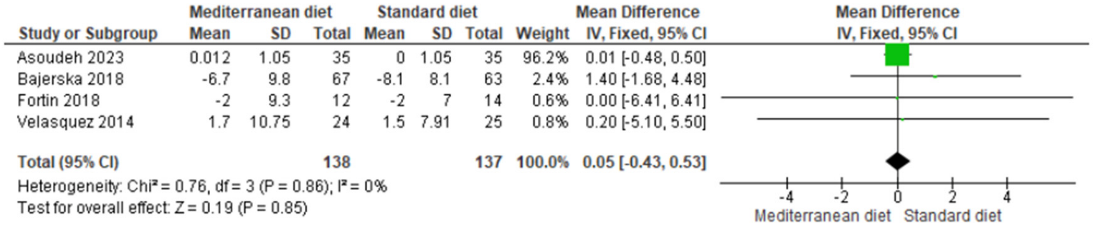

Supplementary Figure S4. Glucose without Esposito et al., 2004

Study or Subgroup	Mediterranean diet Mean	SD	Total	Standard diet Mean	SD	Total	Weight	Mean Difference IV, Random, 95% CI
Asoudeh 2023	-29.7	6.25	35	0.5	6.25	35	46.2%	-30.20 [-33.13, -27.27]
Bajerska 2018	-33.9	88.32	67	-38.8	54.87	63	34.0%	4.90 [-20.22, 30.02]
Velasquez 2014	-90	91.35	24	-30.5	80.9	25	19.7%	-59.50 [-107.89, -11.11]
**Total (95% CI)**			**126**			**123**	**100.0%**	**-24.03 [-52.36, 4.29]**

Heterogeneity: $Tau^2 = 449.46$; $Chi^2 = 8.85$, df = 2 (P = 0.01); $I^2 = 77\%$
Test for overall effect: Z = 1.66 (P = 0.10)

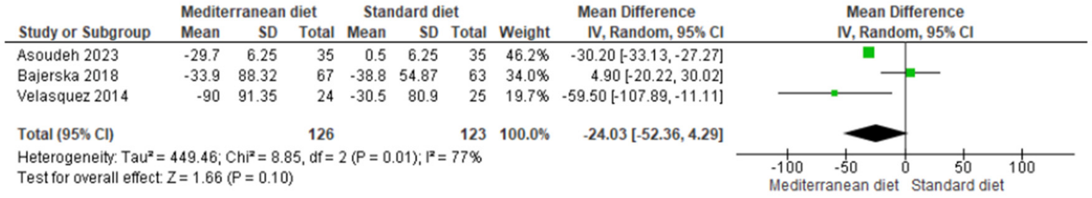

Supplement: Supplementary file 1 [file ijms-26-05887-s001.zip › ijms-3657148-supplementary-figure.pdf]
