# Supplementary material for: Comparison of the Mediterranean Diet and Other Therapeutic Strategies in Metabolic Syndrome: A Systematic Review and Meta-Analysis
Source: Int J Mol Sci. 2025 Jun 19;26(12):5887. doi: 10.3390/ijms26125887 (PMC12192665; doi:10.3390/ijms26125887)
Supplement: Supplementary file 1 [file ijms-26-05887-s001.zip › ijms-3657148-supplementary-table.pdf]

Supplementary Table S1: Details of the search strategy.

Database	Search strategy	Results	
		20-10-24	17-04-2025
Medline	Mediterranean diet AND metabolic syndrome Filters: Clinical Trial, Randomized Controlled Trial (("diet, mediterranean"[Mesh Terms] OR ("diet"[All Fields] AND "mediterranean"[All Fields]) OR "mediterranean diet"[All Fields] OR ("mediterranean"[All Fields] AND "diet"[All Fields])) AND ("metabolic syndrome"[MeSH Terms] OR ("metabolic"[All Fields] AND "syndrome"[All Fields]) OR "metabolic syndrome"[All Fields])) AND (clinical trial[Filter] OR randomized controlled trial[Filter])	115	157
Wos	Mediterranean diet AND metabolic syndrome Filters: Clinical Trial, Randomized Controlled Trial	44	45
CINAHL	Mediterranean diet AND metabolic syndrome Filters: Clinical Trial, Randomized Controlled Trial	12	12
SCOPUS	Mediterranean diet AND metabolic syndrome Filters: Clinical Trial, Randomized Controlled Trial	35	35
Google scholar	Mediterranean diet AND metabolic syndrome Filters: Clinical Trial, Randomized Controlled Trial	106	107
Embase	Mediterranean diet AND metabolic syndrome Filters: Clinical Trial, Randomized Controlled Trial	15	15
	Total	367	370

* All searches were carried out on March 01, 2025.

Supplementary Table S2: PRISMA

Section and Topic	Item #	Checklist item	Location where item is reported
		**TITLE**	
Title	1	Identify the report as a systematic review.	Title page
		**ABSTRACT**	
Abstract	2	See the PRISMA 2020 for Abstracts checklist.	Yes
		**INTRODUCTION**	
Rationale	3	Describe the rationale for the review in the context of existing knowledge.	Pages 2-3
Objectives	4	Provide an explicit statement of the objective(s) or question(s) the review addresses.	Pages 4
		**METHODS**	
Eligibility criteria	5	Specify the inclusion and exclusion criteria for the review and how studies were grouped for the syntheses.	Pages 4
Information sources	6	Specify all databases, registers, websites, organisations, reference lists and other sources searched or consulted to identify studies. Specify the date when each source was last searched or consulted.	Page 5
Search strategy	7	Present the full search strategies for all databases, registers and websites, including any filters and limits used.	Page 5
Selection process	8	Specify the methods used to decide whether a study met the inclusion criteria of the review, including how many reviewers screened each record and each report retrieved, whether they worked independently, and if applicable, details of automation tools used in the process.	Page 4-5
Data collection process	9	Specify the methods used to collect data from reports, including how many reviewers collected data from each report, whether they worked independently, any processes for obtaining or confirming data from study investigators, and if applicable, details of automation tools used in the process.	Pages 6
Data items	10a	List and define all outcomes for which data were sought. Specify whether all results that were compatible with each outcome domain in each study were sought (e.g., for all measures, time points, analyses), and if not, the methods used to decide which results to collect.	Pages 6
	10b	List and define all other variables for which data were sought (e.g., participant and intervention characteristics, funding sources). Describe any assumptions made about any missing or unclear information.	Pages 6
Study risk of bias assessment	11	Specify the methods used to assess risk of bias in the included studies, including details of the tool(s) used, how many reviewers assessed each study and whether they worked independently, and if applicable, details of automation tools used in the process.	Page 6
Effect measures	12	Specify for each outcome the effect measure(s) (e.g., risk ratio, mean difference) used in the synthesis or presentation of results.	Pages 6
Synthesis methods	13a	Describe the processes used to decide which studies were eligible for each synthesis (e.g., tabulating the study intervention characteristics and comparing against the planned groups for each synthesis (item #5)).	Page 6
	13b	Describe any methods required to prepare the data for presentation or synthesis, such as handling of missing summary statistics, or data conversions.	Pages 6
	13c	Describe any methods used to tabulate or visually display results of individual studies and syntheses.	Pages 6
	13d	Describe any methods used to synthesize results and provide a rationale for the choice(s). If meta-analysis was performed, describe the model(s), method(s) to identify the presence and extent of statistical heterogeneity, and software package(s) used.	Pages 6
	13e	Describe any methods used to explore possible causes of heterogeneity among study results (e.g., subgroup analysis, meta-regression).	Pages 7
	13f	Describe any sensitivity analyses conducted to assess robustness of the synthesized results.	Pages 7-8
Reporting bias assessment	14	Describe any methods used to assess risk of bias due to missing results in a synthesis (arising from reporting biases).	Page 7-8

Section and Topic	Item #	Checklist item	Location where item is reported
Certainty assessment	15	Describe any methods used to assess certainty (or confidence) in the body of evidence for an outcome.	Page 7-8
**RESULTS**			
Study selection	16a	Describe the results of the search and selection process, from the number of records identified in the search to the number of studies included in the review, ideally using a flow diagram.	Page 9
	16b	Cite studies that might appear to meet the inclusion criteria, but which were excluded, and explain why they were excluded.	Page 9
Study characteristics	17	Cite each included study and present its characteristics.	Page 10
Risk of bias in studies	18	Present assessments of risk of bias for each included study.	Page 11
Results of individual studies	19	For all outcomes, present, for each study: (a) summary statistics for each group (where appropriate) and (b) an effect estimate and its precision (e.g., confidence/credible interval), ideally using structured tables or plots.	Supplemental Table 1
Results of syntheses	20a	For each synthesis, briefly summarise the characteristics and risk of bias among contributing studies.	Page 11
	20b	Present results of all statistical syntheses conducted. If meta-analysis was conducted, present for each the summary estimate and its precision (e.g., confidence/credible interval) and measures of statistical heterogeneity. If comparing groups, describe the direction of the effect.	Supplemental Tables 1 and 3
	20c	Present results of all investigations of possible causes of heterogeneity among study results.	Supplemental Figures 3 to 8
	20d	Present results of all sensitivity analyses conducted to assess the robustness of the synthesized results.	Supplemental Figures 3 to 8
Reporting biases	21	Present assessments of risk of bias due to missing results (arising from reporting biases) for each synthesis assessed.	Supplemental Table 3
Certainty of evidence	22	Present assessments of certainty (or confidence) in the body of evidence for each outcome assessed.	Supplemental Figures 3 to 8
**DISCUSSION**			
Discussion	23a	Provide a general interpretation of the results in the context of other evidence.	Pages 10 to 14
	23b	Discuss any limitations of the evidence included in the review.	Pages 11 and 12
	23c	Discuss any limitations of the review processes used.	Pages 12 and 13
	23d	Discuss implications of the results for practice, policy, and future research.	Pages 12 to 14
**OTHER INFORMATION**			
Registration and protocol	24a	Provide registration information for the review, including register name and registration number, or state that the review was not registered.	N/A
	24b	Indicate where the review protocol can be accessed, or state that a protocol was not prepared.	N/A
	24c	Describe and explain any amendments to information provided at registration or in the protocol.	N/A
Support	25	Describe sources of financial or non-financial support for the review, and the role of the funders or sponsors in the review.	Page 15
Competing interests	26	Declare any competing interests of review authors.	Page 15
Availability of data, code and other materials	27	Report which of the following are publicly available and where they can be found: template data collection forms; data extracted from included studies; data used for all analyses; analytic code; any other materials used in the review.	Methods, 2.3 Electronic search

From: Page MJ, McKenzie JE, Bossuyt PM, Boutron I, Hoffmann TC, Mulrow CD et al. The PRISMA 2020 statement: an updated guideline for reporting systematic reviews. BMJ 2021;372:n71. doi: 10.1136/bmj.n71

For more information, visit: http://www.prisma-statement.org/

Supplementary Table S3: GRADE

Supplemental Digital Content. Table S3. Summary of Findings (SoF) and quality of evidence (GRADE) for Acid alpha lipoic in patients with neuropathic pain associated with diabetes mellitus.

Certainty assessment							№ of patients		Effect		Quality of evidence (GRADE)	Importance
№ of studies	Study design	Risk of bias	Inconsistency	Indirectness	Imprecision	Other considerations	MD	Control group	Relative (95% CI)	MD (95% CI)		
**Body weight in k/g**												
**5**	RCT	Not Serious	Serious	Not serious	Serious	None	228	227	-	-0.92 (-2.70 to 0.87)	⊕◯◯◯ Very low	CRITICAL
**BMI in kg/m²**												
4	RCT	Not Serious	Very serious	Not serious	Serious	None	161	164	-	-0.83 (-0.93 to -0.74)	⊕◯◯◯ Very low	CRITICAL
**Circumference in cm**												
5	RCT	Not serious	Very serious	Not serious	Serious	None	228	227	-	-1.81 (-2.63 to -0.99)	⊕⊕◯◯ Low	CRITICAL
**SBP**												
5	RCT	Not Serious	Very serious	Not serious	Serious	None	228	227	-	-3.33 (-6.92 to 0.26)	⊕⊕◯◯ Low	IMPORTANT
**DBP**												
**5**	**RCT**	**Not serius**	**Serius**	**Not serious**	**Serious**	**None**	**228**	**227**	**-**	**-0.47, (-2.12 to 1.17)**	**⊕⊕◯◯ Low**	**IMPORTANT**
**Triglycerides**												
**4**	**RCT**	**Serious**	**Serious**	**Not serious**	**Serious**	**None**	**216**	**213**	**-**	**-22.38 (-32.86 to -11.90)**	**⊕◯◯◯ Very low**	**IMPORTANT**
**Glucose**												
**3**	**RCT**	**Serious**	**Serious**	**Not serious**	**Serious**	**None**	**181**	**178**	**-**	**-4.28, (-7.64 to –0.93)**	**⊕◯◯◯ Very low**	**IMPORTANT**

Cholesterol

5	RCT	Serious	Serious	Not serious	Serious	None	228	227		-5.91, (-16.95 to 4.44)	⊕⊕○○ Low	IMPORTANT

HDL

5	RCT	Serious	Serious	Not serious	Serious	None	228	227	-	2.59 (0.64 to 4.55)	⊕○○○ Very low	IMPORTANT

LDL

4	RCT	Serious	Serious	Not serious	Serious	None	138	137	-	-7.96, (-17.06 to -1.14)	⊕○○○ Very low	IMPORTANT

Body fat in kg

2	RCT	Serious	Serious	Not serious	Serious	None	36	39	-	-0.43, (-6.38 to 5.52)	⊕⊕○○ Low	IMPORTANT

HOMA IR

3	RCT	Serious	Serious	Not serious	Serious	None	192	188	-	-0.62, (-1.14 to -0.09)	⊕○○○ Very low	IMPORTANT

Insulin

3	RCT	Serious	Serious	Not serious	Serious	None	192	188	-	-2.21, (-3.90 to -0.52)	⊕⊕○○ Low	IMPORTANT

SMD: Standard Mean Difference; **RCT:** Randomized clinical trial; **Quality of evidence:** High: The research provides a very good indication of the likely effect. The probability that the effect is different is low. Moderate: The research provides a good indication of the likely effect. The probability that the effect is substantially different is moderate. Low: The research gives some indication of the probable effect. However, the probability that the effect is substantially different is high. Very low: The research does not provide a reliable estimate of the probable effect. The probability that the effect is substantially different is very high. **Downgrading:** GRADE approach has four reasons for possible rate down of the quality of evidence. Begins with the study designs (trials or observational studies), secondly downgrading the evidence one level (1) for study limitation if the majority of studies (>50%) were rated as high risk of bias; (2) for inconsistency, if heterogeneity was greater than the accepted low level (I^2 >40%); (3) for indirectness, directness was undoubled; (4) for imprecision, if meta-analysis had a small sample size (n <400) or the confidence interval very wide.
